# Counting on Potential Grandparents? Adult Children’s Entry Into Parenthood Across European Countries

**DOI:** 10.1007/s13524-020-00890-8

**Published:** 2020-06-09

**Authors:** Roberta Rutigliano

**Affiliations:** Population Research Centre, Faculty of Spatial Sciences, University of Groningen, Landleven, 1 -9747, Groningen, AD the Netherlands

**Keywords:** Childcare, Transition to parenthood, Fertility, Grandparents, Intergenerational relations

## Abstract

As populations age and longevity rises, the structure of the extended family is changing. Parents of young children are increasingly turning to the children’s grandparents to provide childcare and help them reconcile work and family. This study is the first to investigate whether would-be grandparents’ propensity to care for their grandchildren influences the adult children’s transition to parenthood. Because grandparental childcare provision is not observable at the time of the transition to the first birth, I built a measure based on the characteristics of both actual grandparents and adult children to act as a proxy for the childcare that prospective grandparents are expected to provide in the future. Using data from the first two waves of the Survey of Health, Aging, and Retirement in Europe, I examine changes in the likelihood of having a first birth by different levels of expected future childcare provision. Given that the role grandparents play varies depending on the national context, I estimate distinct models for different groups of countries. Furthermore, I analyze different intensities of grandparental childcare: regular, occasional, and any other type of positive childcare. The comparison across 11 countries reveals that grandparental propensity to provide occasional childcare has a positive effect on the transition to parenthood in all country clusters and that grandparental propensity to provide regular childcare has a positive and significant association with having a first child in both pro-natalist (Belgium and France) and pro-traditional countries (Austria, Germany, Greece, Italy, Spain, and Switzerland).

**Electronic supplementary material:**

The online version of this article (10.1007/s13524-020-00890-8) contains supplementary material, which is available to authorized users.

## Introduction

Parents are among the most important sources of emotional and material support for their adult children. In light of the increase in both age at first birth and longevity, parents are more likely than in the past to live for many years while their children are adults. Thus, today’s parents are in a better position than previous generations to assume an important role in the lives of their children and grandchildren (Bengtson 2001; Bengtson et al. 1990; Chapman et al. 2018; Gauthier 2002; Margolis and Wright 2017; Silverstein 2005). When it comes to fertility, parental characteristics might materially influence the fertility outcomes of their adult children. When the parents become grandparents, they often represent the main affordable and flexible source of informal childcare for their children, and they may serve as crucial facilitators of work-family balance (Arpino et al. 2014; Fergusson et al. 2008).

Most research on the impact of grandparental support on the fertility of adult children has focused on the transition from first to second or subsequent births (e.g., Aassve et al. 2012b; Tanskanen and Rotkirch 2014; Thomese and Liefbroer 2013). Studies investigating the influence of parental support (i.e., in-time or in-money transfers) on adult children’s entry into parenthood are scarce. One of the main reasons for this research gap is that (grand)parental childcare provision is observable only after childbirth. Nonetheless, there are several reasons for studying the first-birth transitions of adult children. First, entry into parenthood is a crucial event in an individual’s life (Rindfuss et al. 2007): it dramatically affects the individual’s happiness and organization of daily life (Margolis and Myrskylä 2011; Myrskylä and Margolis 2014). The decision to have a first child is complex. It is more affective and less rational than other fertility transitions, yet it represents the onset of the family-building process (Morgan 2003). A positive experience at the transition to parenthood is likely to increase the odds of subsequent births (Margolis and Myrskylä 2015; Newman 2008). At a time as unique as the transition to parenthood, adult children’s perceptions and expectations of having their own children might also be influenced by the level of involvement they can expect from their parents as grandparents.

In this article, I argue that although first-time parents cannot predict how much support they will receive from their own parents, they can anticipate future levels of grandparental childcare provision by observing their parents’ behaviors and characteristics, and adjust their decision to enter parenthood accordingly. Therefore, individual characteristics of would-be grandparents may be expected to jointly influence future childcare provision. The theoretical strength and the novelty of this approach lie in taking this multidimensionality into account. The few previous quantitative studies that investigated the interplay between family formation and parental influence focused on a single grandparental characteristic, such as geographical distance (e.g., Pink2018) or the physical presence of grandparents (for detailed discussions, see Mathews and Sear 2013; Sear and Coall 2011; Tanskanen and Danielsbacka 2019). Yet, as Aassve and his colleagues explained (2012b), the mere availability of grandparents is not enough to serve as a proxy for grandparental childcare provision. (Grand)parents also need to be active and willing to provide childcare. In some cases, despite living close to their adult children, grandparents may be unable to look after (grand)children due to ill health or old age. In this article, I try to incorporate this complexity into the model by building a measure for the caring potential of would-be grandparents that considers several characteristics of prospective grandparents simultaneously.

Individual decisions are influenced by social interactions and cultural factors (Montgomery and Casterline 1996; Rosero-Bixby and Casterline 1994). Specifically, whether a parent prefers one type of care to another depends on the interplay of cultural values, family models, and public policies (Pfau-Effinger 2005). Thus, the relative importance of grandparental care and the ways in which individuals divide family responsibilities in different countries may lead to the emergence of distinct scenarios (Arber and Timonen 2012). A second contribution of the current study is that I explore the macro-level heterogeneity associated with differences in regional perceptions of the grandparental role (for a discussion across European countries, see Jappens and Van Bavel 2012).

I address two research questions. First, under what conditions is the transition to parenthood among adult children influenced by the characteristics of their parents as potential grandparents? Second, to what extent does this relationship vary across groups of countries? The contribution of this study to the existing literature is twofold. First, I look specifically at how the individual characteristics of the would-be grandparents—interpreted as indicators of their propensity to provide childcare—affect the entry into parenthood of their adult children. By focusing on the first-birth transition, this study contributes to the literature on the influence of social support on family formation. Second, to account for the interplay between institutional contexts and individual fertility behaviors, I use a cross-national comparative design to carry out distinct analyses for different groups of countries. Furthermore, I look at different intensities of grandparental provision of childcare (i.e., regular, occasional, and any type) because the trade-off between the prevalence and the intensity of grandparental childcare varies across contexts (Gauthier 2002; Hank and Buber 2009; Herlofson and Hagestad 2012; Jappens and Van Bavel, 2012).

## Background

### The Influence of Grandparents on Fertility at the Micro Level

The role of (grand)parents in fertility transitions has been studied in a wide range of disciplines, including economics, sociology, and evolutionary biology. Substantial evidence suggests that (grand)parents are far more likely than other relatives and friends to provide their adult children with help in the form of time, money, and emotional support (for overviews, see Coall and Hertwig 2010, 2011; Fergusson et al. 2008; Sear and Coall 2011). In evolutionary biology, the inclusive fitness theory (Hamilton 1964) states that parents are often willing to take care of the offspring of their adult children in order to increase the survival probability of their lineage. Extending this biological argument to grandparents, one could argue that maternal grandmothers, who are certain to share lineage with their grandchildren, have a greater interest in looking after their kin than grandfathers, who may or may not be biologically related to the grandchildren (for detailed discussions, see Mathews and Sear 2013; Tanskanen and Danielsbacka 2019).

In sociology and economics, researchers often posit that parents influence the fertility transitions of their adult children via intergenerational transfers. Further, individuals are assumed to base their decisions about the number and the timing of their births on a cost-benefit analysis (Becker 1981). Parental transfers might soften the perceived costs of parenthood for adult children. According to Liefbroer (2005), when women were asked to identify the costs related to childbirth, they were most likely to cite the substantial decline in both their career opportunities and individual autonomy. It is, therefore, likely that having parental support—and help with childcare in particular—lowers these costs. This hypothesis is empirically well grounded. Research has found that grandparents provide a considerable amount of care for their grandchildren (Aassve et al. 2012b; Gauthier 2002; Mathews and Sear 2013; Thomese and Liefbroer 2013) and that this support positively affects the labor force participation and subsequent fertility transitions of their adult children, particularly of their daughters (Aassve et al. 2012a,b; Thomese and Liefbroer 2013).

At the micro level, the literature has mainly focused on the parental characteristics that influence an adult child’s fertility dynamics. In this framework, grandparental childcare provision has been shown to have a positive impact on an adult daughter’s second and subsequent fertility transitions (e.g., Aassve et al. 2012b; Thomese and Liefbroer 2013). However, just as grandparental support has been found to enhance work-life balance for women (Aassve et al. 2012a), the increased labor force participation of women has been shown to stimulate grandparental childcare provision (Geurts et al. 2009; Gray 2005).

Geographical proximity between the two generations is especially important: grandparents and their adult children need to live close to each other to allow the grandparents to provide childcare (Compton and Pollak 2014; Heylen et al. 2012). Beyond the current availability of grandparental childcare, geographical proximity can influence an adult child’s future expectations of receiving childcare support and can thus create anticipation mechanisms; the closer the parents live to their adult child, the greater the adult child’s expectations for future support, and the higher the adult child’s propensity for having a first or a second birth (Heylen et al. 2012; Pink 2018).

Gender is another factor that shapes the nature of grandparental childcare provision. In line with traditional gender roles—and possibly due to factors related to evolutionary biology—grandmothers are typically more involved in caring for their grandchildren than grandfathers, especially when the childcare intensity is high (Hank and Buber 2009; Wheelock and Jones 2002) and the grandfather is still working (Kahn et al. 2011). A grandparent’s age has a negative impact on the likelihood of providing childcare: the older the grandparent is, the higher the likelihood of morbidity, and the lower the level of support he or she is able to provide (Aassve et al. 2012b; Kaptijn et al. 2010; Thomese and Liefbroer 2013). Finally, the higher the number of grandparents who are active in an adult child’s life, the greater the likelihood that the adult child will have an additional birth (Thomese and Liefbroer 2013). A grandparent who is employed is more time-constrained. On the other hand, Van Bavel and De Winter (2013) showed that becoming a grandparent may accelerate retirement, especially among women.

Two main gaps emerge in this strain of sociodemographic research. First, most studies have focused on the effect of grandparental childcare on an adult child’s second or higher-order births. Second, most of these studies considered grandparental characteristics separately.

### The Interaction Between the Role of Grandparents and the Type of Context

The influence of parents on the fertility of their adult children might vary across different care regimes and contexts (Arber and Timonen 2012; Jappens and Van Bavel 2012; Pfau-Effinger 2004). According to Herlofson and Hagestad (2012), two main dynamics are at play in this association. First, in places where there is a universal public childcare system, grandparents tend to help their adult daughters, especially if they are working and primarily when intensive childcare is required. For example, even if public childcare services are available, grandparental support may be needed if a grandchild has a disability. Families may also rely on the informal support of grandparents in unexpected situations or in cases in which a child needs a level of care that public childcare services cannot fully provide. Second, in places with a familialistic welfare system, grandparents may agree to help raise their grandchildren because of a lack of public childcare. Herlofson and Hagestad (2012) observed that in the first context, grandparents are “family savers” who add to the flexibility of an already efficient system by providing extra childcare; in a familialistic regime, grandparents are “mother savers” who perform services not provided by the welfare state.

Cultural values, together with new family policies and emerging social roles, can also shape families’ childcare arrangements (Arber and Timonen 2012). First, as Jappens and Van Bavel (2012) underlined, the normative climate often leads mothers to prefer a specific type of childcare. Mothers who live in a traditional context tend to prefer grandparental to formal childcare, whereas mothers who live in a more progressive context tend to prefer formal to grandparental childcare (Jappens and Van Bavel 2012). Second, cultural values can influence grandparents’ perceptions of their roles and responsibilities. By combining Norwegian register data and survey data from other European countries, Herlofson and Hagestad (2012) showed general agreement across countries that grandparents are supportive figures. However, national differences emerge when the types of support grandparents are expected to provide are decomposed. For instance, approximately 35% of grandparents in southern Europe but less than 10% of grandparents in Denmark and the Netherlands strongly believe that their support should also involve an economic dimension (Herlofson and Hagestad 2012).

Hank and Buber (2009) also found an inverse relationship between the prevalence and the intensity of grandparental childcare provision across European countries. In countries with stronger family norms, such as Spain and Italy, the percentage of grandparents who were providing some childcare was shown to be particularly low. However, when the authors distinguished between regular and occasional childcare, they found that those countries have above-average levels of grandparental childcare provision because the amount of regular care that was provided was relatively high. In Scandinavia, where family norms are weaker, the authors found that the share of grandparents who had provided some childcare over the year was high but that the amount of regular childcare provided was below average (Hank and Buber 2009).

Finally, another potential source of contextual variation is the social perception of childlessness. In Europe, and especially in the Nordic and Western countries, a higher prevalence of individualistic values is associated with a greater acceptance of child-free lifestyles (Merz and Liefbroer 2012). However, the role of attitudes toward childlessness in fertility is difficult to disentangle. We can expect (grand)parental influence to be lower in countries with less favorable attitudes toward childlessness because adult children there are likely to have a first child regardless of the availability of grandparental support. If this is the case, these countries should have the highest percentage of first births. However, this relationship appears to be more complicated. For example, compared with other European countries, Italy has the lowest social acceptance of childlessness (Merz and Liefbroer 2012; Sobotka and Testa 2008) but a high percentage of childless women (Beaujouan et al. 2017; ISTAT 2018). Childlessness might be the result of a continuous postponement of parenthood (Rybińska and Morgan 2019), which may in turn be attributed to a lack of resources, weak family policies, and/or adjustments of expectations (Beaujouan et al. 2017; Rybińska and Morgan 2019; Sobotka and Testa 2008). Thus, it is also possible that in countries where social acceptance of childlessness is low and family policies are weak, the availability of grandparental childcare plays an even larger role in an adult child’s decision to become a parent.

### European Country Clusters

**Fig. 1:**
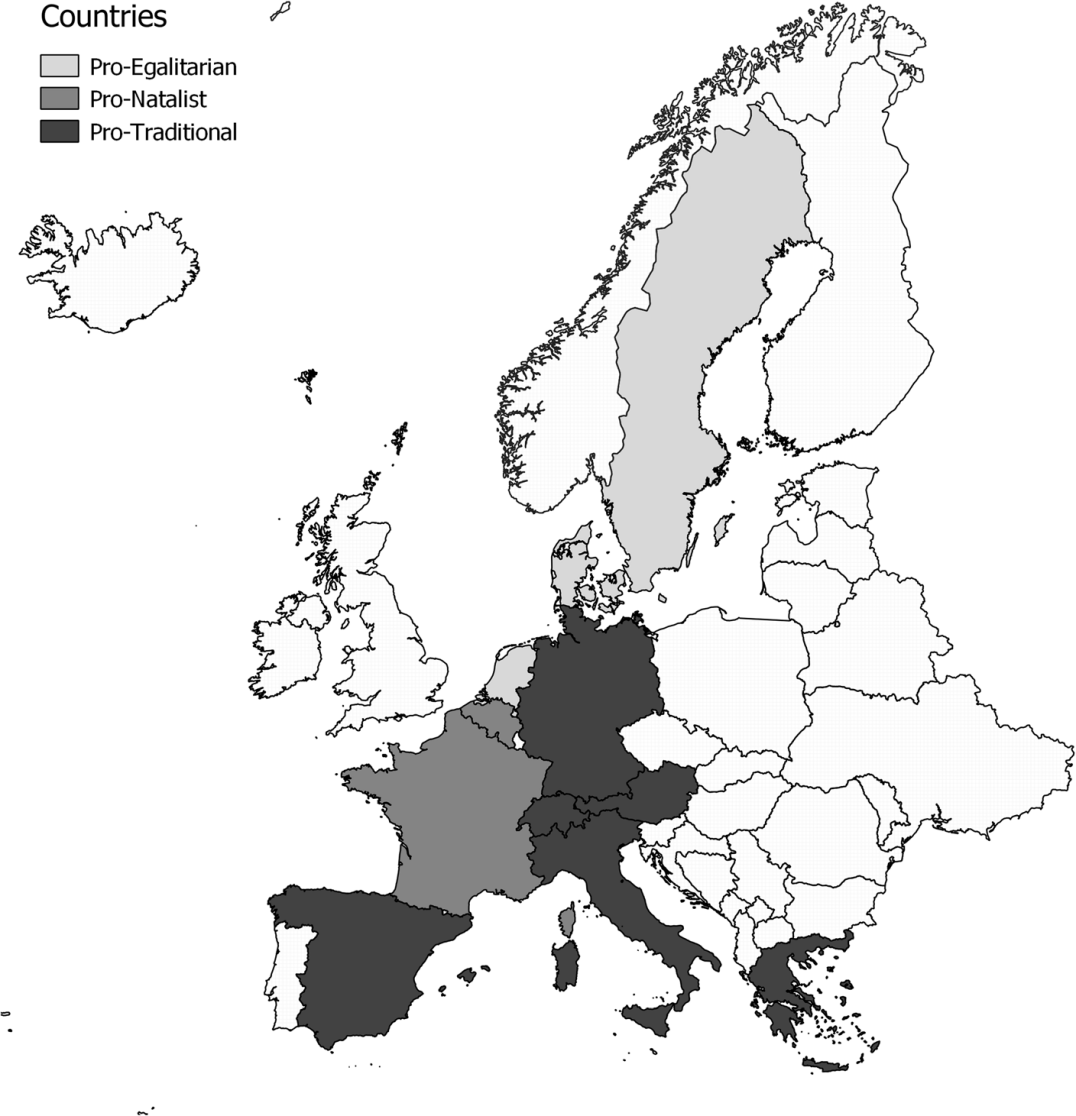
Group of countries according to Gauthier’s classification

Several classification systems have been offered for comparing nation-specific institutional characteristics (e.g., Esping-Andersen 1990). Given the aims of this study, I decided to use a classification system that considers both social norms and institutions, with a particular focus on childcare provision: namely, Gauthier’s (1996) family regime typology (see Fig. 1). In this typology, countries are grouped along the two dimensions of family policies and social values. One of the main advantages of Gauthier’s classification is that it is childcare-specific. Although some policies aimed at caregivers might be very generous on average, a careful breakdown of these policies may show that they mainly support care at older ages and do not cover childcare, or vice versa (e.g., Albertini et al. 2007). For this reason, Gauthier’s classification system is particularly suitable for use in studies on childcare and fertility (e.g., Del Boca et al. 2009).

By considering family policies and social values simultaneously, Gauthier’s classification captures the reaction of different regimes to the decline of the breadwinner model and the impact of this decline on fertility. Because an increase in women’s paid work reduces the time mothers can spend caring for their children, governments should fill this care gap. Countries are assigned to clusters based on both the responsiveness of public policies—which, in turn, depends on family norms—and the types of responses. Gauthier (1996) included measures such as levels of cash benefits, maternity leave, provision of childcare facilities, and the openness of abortion laws. Placing each country squarely within a single group, of course, is difficult. All the countries considered here have experienced a modernization process and several reforms of public policies. Thus, my goal is to identify the major policy differences that persist over time (Thévenon 2011).

Gauthier (1996) identified four regimes: pro-traditional (in my sample, Austria, Germany, Greece, Italy, Spain, and Switzerland), pro-egalitarian (in my sample, Denmark, the Netherlands, and, Sweden), pro-family/pro-natalist (in my sample, Belgium and France), and pro-family but noninterventionist (the United Kingdom and Australia, which are not in my sample and are not discussed further). Pro-traditional countries shown an asymmetry between the strong emphasis placed on the family and actual family policies, which may be fairly unsupportive. The levels of support provided for family formation by the welfare states in these countries are not in line with the needs of the citizens. In these countries, approximately 50% of grandparents provide at least occasional childcare; and of this group, between 20% and 50% provide daily childcare (Herlofson and Hagestad 2012). A RAND Europe dossier (Mills et al. 2014) reported that in pro-traditional countries (not including Spain) in 2010, the percentage of children under age 3 in formal childcare arrangements varied from almost 6% in Greece to about 28% in Switzerland. The study also found that the percentage of children under age 3 in informal childcare was particularly high in these countries: excluding Germany, the share ranged from 30% to 60%. Thévenon (2011) found that the maternal employment rate for women with children under age 2 in these countries was between 47.3% in Italy and 60.5% in Austria.

In the pro-natalist countries of France and Belgium, both policies and norms support family formation. According to Gauthier (1996), in this cluster of countries, both family formation and fertility are seen as important goals. Childcare coverage is much higher in these countries than in other continental European countries (Thévenon 2011). The percentage of children under age 3 in formal childcare arrangements is above the Barcelona target for both countries: namely, approximately 40% in Belgium and approximately 45% in France (Mills et al. 2014). Moreover, the employment rate for mothers with children under age 2 is fairly high, at 63.8% for Belgium and 53.7% for France (Thévenon 2011). At the same time, however, family values are well established in these countries. The percentage of children under age 3 in informal childcare fluctuates at roughly 20% (Mills et al. 2014). Moreover, between 40% and 60% of grandparents in France and Belgium strongly agree that it is their duty to be there for their grandchildren in case of difficulties (Herlofson and Hagestad 2012). Finally, among the 60% of grandparents who look after their grandchildren at least occasionally, the percentage providing almost daily childcare is approximately 20% (Herlofson and Hagestad 2012). These percentages are lower than in the pro-traditional countries but are higher than in the pro-egalitarian countries.

In the pro-egalitarian countries (Denmark, the Netherlands, and Sweden), gender equality is prioritized. The welfare state provides a high level of assistance for achieving work-family balance (Gauthier 1996). In this cluster, more than 65% of grandparents provide at least occasional childcare. However, just 5% of these grandparents provide care on an almost daily basis—the lowest share in Europe (Herlofson and Hagestad 2012). The percentage of children under age 3 who are in informal childcare provided by grandparents or others is almost 0 in Denmark and Sweden. The percentage of children under age 3 in formal childcare arrangements is roughly 50% in the Netherlands and Sweden but is close to 80% in Denmark (Mills et al. 2014). Moreover, the maternal employment rate for women with children under age 2 ranges from 69% in the Netherlands to 72% in Denmark (Thévenon 2011). In these countries, “instead of promoting a traditional family, the main concern has been the achievement of a more egalitarian sex-role model. [. . .] Legislation on parental leave, as opposed to maternal leave, has been one of the centre-pieces of this model” (Gauthier 1996:204).

The data used for the analysis do not include countries that are classified as pro-family but noninterventionist. Therefore, the rest of the article will focus on the three regimes covered in my analysis: pro-egalitarian, pro-traditional, and pro-natalist.

## Hypothesis

For each country cluster, I consider two types of grandparental support: regular support provided at least once per week, and occasional support provided less than once per week.^1^ Regular grandparental childcare provision is more intense. In contexts where this type of childcare is more prevalent, grandparents are “mother savers” (Herlofson and Hagestad 2012). Occasional grandparental childcare provision is less intense but still very useful. In these contexts, grandparents are “family savers” (Herlofson and Hagestad 2012).

In the pro-traditional countries, I expect to find that both types of expected grandparental childcare propensity have a positive impact on the likelihood of an adult child having a first child. Two mechanisms can be identified in this context. First, because family obligations and social roles are relatively strong in these countries, grandparents may agree to take care of their grandchildren because they feel entitled to do so and because they want to be socially perceived as good grandparents. Building on previous studies, the prospective grandparents in pro-traditional countries are expected to be more responsive to their adult children’s needs than their counterparts in other countries (e.g., Daatland et al. 2011; Herlofson and Hagestad 2012; Kalmijn and Saraceno 2008). Second, based on prior research, I assume that adult children in these countries can be assumed to prefer to rely fully on informal care because they see it as the norm and believe that it offers the best outcomes and highest degree of reliability (Arpino et al. 2014). Taken together, the presence of these two mechanisms implies that welfare state services play a marginal role in fertility decisions. Indeed, pro-traditional countries have been found to have the highest intensities in terms of downward transfers and grandparental childcare provision (Albertini et al. 2007; Hank and Buber 2009). By contrast, in the more individualistic pro-egalitarian countries, where the welfare state is more supportive and parents mainly rely on formal childcare, I expect to find that grandparental childcare provision matters less. I also assume that in the pro-natalist countries, where individuals rely on both the welfare state and the family, the availability of grandparental childcare does not play a decisive role in fertility.

## Methods

The data used in the analysis are from the Survey of Health, Aging, and Retirement in Europe (SHARE http://www.share-project.org/; Börsch-Supan 2016). SHARE is a cross-national European panel data set that contains information on aspects of the living conditions of the older population (aged 50+). Six waves, from 2004/2005 to 2013, are currently available, and data are collected every two years. A clear advantage of this data set is that it includes information that older people have provided about their adult children. Thus, for each respondent, I have fairly detailed information on the demographic characteristics of their children, including fertility behavior of up to four of their adult children.

I apply a two-step approach, using a different sample at each stage. In the first step, I draw on a sample of actual grandparents. I estimate a linear probability model that measures actual grandparental childcare propensity according to both the actual grandparents’ and the adult child’s characteristics. In the second step, I draw on a sample of would-be grandparents, and use the results from the first step to predict the future grandparental childcare propensity of these would-be grandparents. The reason for this research design is twofold. First, because my focus is on first-birth transitions, actual grandparental childcare cannot be observed. A proxy for expected future childcare provision is therefore needed. The grandparental childcare provision propensity derived from the first step offers such a measure. From the adult child’s perspective, the propensity of their parents to provide childcare may be seen not only as a proxy for childcare provision but also as a predictor of the care they will have to provide in the future to both their parents and their offspring. Second, by splitting the estimation process into two steps, each on the basis of different samples, I avoid having a unique regression with highly correlated covariates (more details are provided in the online appendix).

To address country-level heterogeneity, I run separate models for the three different groups of countries obtained on the basis of Gauthier’s classification in both the first and the second step.^2^ The models are run for any type of grandparental childcare provision, not just for regular and occasional childcare. This last measure is obtained by combining provisional and regular childcare; thus, I will refer to this model as the *any type of childcare* specification. I present the results for this third specification as well but only as a benchmark.

### First Step

In the first step, I examine how the different circumstances and personal characteristics of adult children and their parents influence the likelihood of grandparental childcare provision. Thus, I select variables that reflect different dimensions of grandparents’ lives: namely, variables that Aassve and his colleagues (2012b) defined as health, availability, and willingness to provide care. As part of the health dimension, I select objective measures of health, such as grip strength (considered a good predictor of vitality; Rantanen et al. 1999), recall ability, verbal fluency, and orientation in time. I also include information on self-perceived health and, as a proxy for mental health, the depression scale. Furthermore, to measure grandparents’ availability and willingness to provide care, I include information about their geographical proximity to their children and their time constraints by drawing on data on social and voluntary activities available in SHARE. Finally, I include basic demographic information about both the grandparents and the adult children (i.e., sex and age).

Because health, availability, and willingness to provide childcare are intertwined, they can be difficult to differentiate. For instance, grandparents’ involvement in voluntary and social activities might signal that they are less available because they will have less spare time to spend providing care. Conversely, grandparents’ engagement in social activities could indicate that they have a more active lifestyle, which would be positively correlated with active grandparenting (Arpino and Bordone 2014; Waldrop and Weber 2001) and therefore with willingness to provide care. Table A10 in the online appendix, provides an overview of the grandparental characteristics I include in the model and their associated mechanism(s).

The sample is drawn from the cross-sectional sample of the first wave (i.e., 2004/2005) of SHARE. I select all the individuals from the oldest generation with at least one grandchild regardless of whether they provide childcare. This is the most important selection criterion that I apply because it leads to about 27% of the households being dropped. The final pooled sample consists of 13,330 actual grandparents representing three country groups: 4,520 in pro-egalitarian countries, 5,140 in pro-traditional countries, and 3,670 in pro-natalist countries.

The dependent variable is dichotomous: it equals 1 if a grandparent is providing childcare, and equals 0 otherwise. I run different analyses for different types of grandparental childcare provision. In the first analysis of regular childcare, the dependent variable equals 1 only if childcare is provided at least weekly. In the second analysis of occasional childcare, the dependent variable equals 1 if childcare is provided monthly or less often. In the last analysis, I consider any type of grandparental childcare provision: regular and/or occasional. The need for grandparental support varies according to the context. Thus, considering only one type of childcare would not provide an accurate picture of the role of grandparents in providing childcare. Table 1 shows the dependent variable distribution in the first step for the three model specifications and for the three country clusters. As expected, I find that the pro-traditional countries have the highest levels of regular grandparental childcare, whereas the pro-egalitarian countries have the highest levels of occasional grandparental childcare. The pro-natalist countries lie between these two groups, with higher levels of regular grandparental childcare than the pro-egalitarian countries but lower levels of occasional grandparental childcare than the pro-traditional countries. In all three country clusters, about one-half of the grandparents provide some type of childcare.

**Table 1:** Dependent variable distribution for each group of countries: First step

	Regular Childcare = 1		Occasional Childcare = 1		Any Type of Childcare = 1	
	*N*	%	*N*	%	*N*	%
Pro-Natalist (*N* = 3,670)	876	23.87	1,022	27.85	1,898	51.7
Pro-Traditional (*N* = 5,140)	1,580	30.74	1,054	20.51	2,634	51.2
Pro-Egalitarian (*N* = 4,520)	658	14.56	1,616	35.75	2,274	50.3

*Note: Regular* means that childcare is provided at least once per week, *occasional* means that childcare is provided no more than once per month, and *any type* refers to any positive intensity of childcare provision.

I run a linear probability model to assess the contribution of each variable in determining grandparental childcare provision (regular, occasional, or both). In the second step, I use these contributions to obtain, for each prospective grandparent, a unique predicted measure for the expected grandparent’s propensity to provide childcare. Because of the clustered data structure, whereby the adult child is nested within the parent, the standard errors in the linear probability model are adjusted. For each group of countries, for each adult child *i* nested under his or her parent *f*, the model can be written as follows:

1 $$ {\displaystyle \begin{array}{c}{ChildcareProvision}_{f,i}={\upbeta}_0+{\upbeta}_1{PH}_{f,i}+{\upbeta}_2{CF}_{f,i}+{\upbeta}_3{B}_{f,i}+\\ {}{\upbeta}_4{MH}_{f,i}+{\upbeta}_5{AC}_{f,i}+{\upbeta}_6{GP}_{f,i}+{\upvarepsilon}_{i,f},\end{array}} $$

where *PH* is a set of covariates measuring grandparental physical health, *CF* is a set of variables related to grandparental cognitive function, *B* comprises variables concerning health-related behaviors, *AC* is made up of covariates for the adult children’s characteristics, *GP* is a set of variables for the grandparents’ other characteristics, and *MH* concerns mental health (see the online appendix, section A, for details). I repeat the same model for the three specifications of the dependent variable.

Table A2 in the online appendix shows the coefficients associated with regular grandparental childcare provision for the different grandparental characteristics.^3^ For instance, being employed is significantly and negatively associated with regular grandparental childcare provision (–0.061). Thus, in the second step, adult children with employed parents in *t* – 1,^4^ where *t* is the year of childbirth, score lower on the would-be grandparents’ propensity to provide childcare. All the coefficients from this first step are used in the second step to calculate a predicted score for the prospective grandparents’ childcare provision. All the variables are consistent across the two estimation steps. Additional details on the covariates are provided in section A of the online appendix.

### Second Step

In the second step, I explore the effect of the would-be grandparents’ propensity to provide childcare upon their adult children’s first-birth transition. First, I build a synthetic measure that assigns to each would-be grandparent the likelihood that he or she will provide care for the grandchild.^5^ Second, I assess how this measure influences the first-birth transition. The novelty of this measure, which I call *grandparental childcare propensity* (GP* in upcoming Eq. (2)), is that it condenses different dimensions of childcare. Given that this measure is obtained by using the predictions of the first step’s coefficients on the second step’s sample, it serves as a proxy for expected future childcare provision. The would-be grandparents’ characteristics in *t* – 1 are linked with the actual grandparents’ characteristics. The same approach is used for the adult child’s characteristics. Therefore, for each would-be grandparent, I use all the first step coefficients to predict would-be grandparents’ propensity to provide childcare based on both their own and their adult child’s characteristics in *t* – 1—that is, two years before the birth of the grandchild. The outcome of this prediction becomes the main explanatory variable in the second step (i.e., the grandparental childcare propensity).

In the second step, I rely on the longitudinal sample obtained by merging the first two waves (i.e., 2004/2005 and 2007) for the 11 countries for which I have longitudinal information. Although more waves of SHARE are available, I chose to analyze only the first two because of attrition and data constraints. Specifically, after the first two waves, SHARE released a wave with retrospective pieces of information only (SHARELIFE). In addition, the next wave of longitudinal data (Wave 4) for my sample is available six years after the first wave.

In the second step, I select all the adult children for whom I might observe a first-birth transition between the two waves. More specifically, I select the adult daughters aged 21–45 and the adult sons aged 21–50 (inclusive). I fix the lower bound of the age range at 21 to avoid cases of teen pregnancy, which involve different mechanisms of intergenerational support (Sadler and Clemmens 2004). The upper bound differs by gender because men typically enter parenthood later than women. I also drop individuals for whom the dependent variable is missing (less than 0.001% of the sample). The other controls refer to very basic individual information, so there is no particular bias or selection on missing values (see Table A1 in the online appendix). The overall *N* is 9,258, and the adult children are divided across the different caring regimes as follows: 2,517 in the pro-egalitarian countries, 4,710 in the pro-traditional countries, and 2,031 in the pro-natalist countries (see Table 2).

**Table 2:** Dependent variable distribution (first-birth transitions) for each group of countries: Second step

	Pro-Egalitarian		Pro-Traditional		Pro-Natalist	
	*N*	%	*N*	%	*N*	%
0	2,092	83.2	4,184	88.8	1,768	87.0
1	425	16.8	526	11.2	263	12.9
Total	2,517		4,710		2,031	

*Note:* Here, the dependent variable measures whether a first birth occurred between the two waves.

Although information is reported by the oldest generation, the majority of the adult children are not coresident with the respondents. In line with previous studies (Aassve et al. 2012b), I address the lack of identification numbers for the adult children by matching individuals based on their gender and date of birth. This approach allows me to link information on each adult child across different waves. The dependent variable is derived by combining information across the two waves. It assumes a value equal to 1 if an individual *i*, childless at *t* − 1, experiences a first birth between *t* – 1 and *t*. Table 2 shows the dependent variable distribution. The pro-egalitarian countries have the highest birth numbers, whereas the pro-traditional countries have the lowest fertility levels and the highest childlessness levels. As expected, I find that the fertility patterns of the pro-natalist countries are in the middle, although they are closer to those of the pro-traditional than of the pro-egalitarian countries.

I use a logistic model, adjusting standard errors due to the clustered structure of the data.^6^ The use of the event-history approach was not possible because of data limitations. Formally, for each individual adult child *i* with a parent *f*, the model equation can be written as

2 $$ {\displaystyle \begin{array}{c}{Firstbirth}_{f,\mathrm{i}\left[t-1,t\right]}={\upbeta}_0+{\upbeta}_1{{\mathrm{GP}}^{\ast}}_{f,i,\left(t-1\right)}+{\upbeta}_2 Sex\ {adult\ child}_{f,i,t-1}+\\ {}{\upbeta}_3 Sex\ (grand){parent}_{f,i,t-1}+{\upbeta}_4 Birth\ cohort\ {adult\ child}_{f,i,t-1}+\\ {}{\upbeta}_5 Birth\ cohort\ (grand){parent}_{f,i,t-1}+{\upvarepsilon}_{f,i}.\end{array}} $$

GP* represents the grandparental childcare propensity.^7^ It is computed by linearly predicting the expected childcare provision of would-be grandparents in *t* – 1, based on the first step results—that is, based on the relationship found between the actual grandparental childcare provision and the actual characteristics of the adult children and the grandparents. In the second step, I include a few additional independent variables for the sake of parsimony, especially in light of how the grandparental childcare propensity for providing childcare is estimated. All the covariates are measured at *t* − 1 because this is when individuals might start planning their transition to parenthood.

## Results

### First Step

The results from the linear probability model, in which the dependent variable is whether the actual grandparent provides childcare (i.e., regular, occasional, or any type of childcare), are generally in line with those in the literature (see Tables A2, A4, and A6 in the online appendix). For the three groups of countries, employed grandparents are less likely to provide childcare than retired grandparents. The number of adult children reduces the grandparents’ propensity to provide childcare, and geographical proximity between the grandparents and their adult children increases the likelihood of the grandparents providing childcare, regardless of the country cluster. Specifically, parents who live near their adult children are more likely than coresident parents to provide childcare.^8^ One potential explanation for this finding is that selection into coresidence suggests that the parents may be less healthy and might themselves need care, rather than being in a position to provide it.

### Second Step

**Fig. 2:**
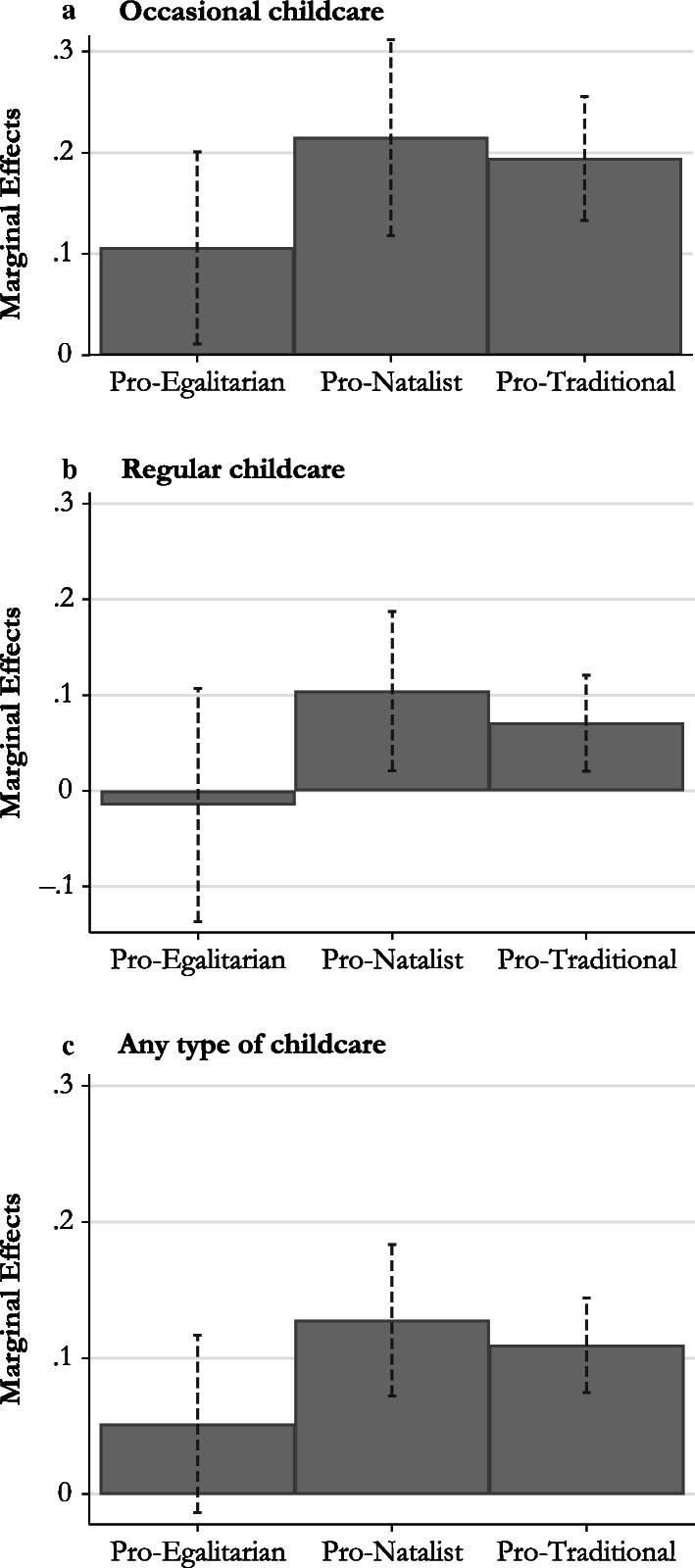
Marginal effect of grandparental childcare propensity on having a first birth by group of country and type of expected childcare provision (second step)

**Fig. 3:**
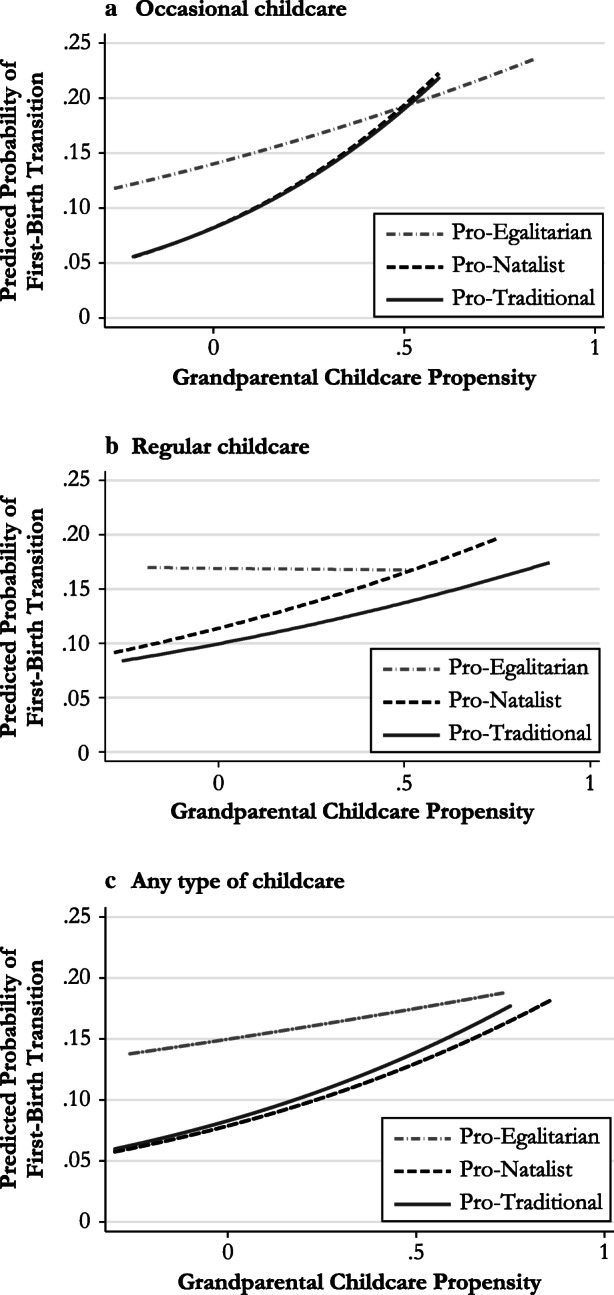
Predicted probability of grandparental childcare propensity on having a first birth by group of countries and type of expected childcare provision (second step)

In this step, for each group of countries and for each type of expected grandparental childcare provision (i.e., regular, occasional, or any type of childcare), I present the marginal effects (Fig. 2) and the predicted probabilities (Fig. 3) of different types of grandparental childcare propensity on having a first birth. Higher values of grandparental childcare propensity correspond to higher levels of expected grandparental childcare provision (complete results in Tables A3, A5, and A7 in the online appendix).

Figure 2 shows the marginal effects of occasional, regular, and any type of grandparental childcare propensity, after adjusting for all other covariates. Looking at both the pro-natalist and pro-traditional countries, we can see that net of all the controls included in the model, the marginal effects are positive and significant in both specifications. In the pro-egalitarian countries, a slightly negative but not statistically significant effect of regular grandparental childcare propensity is observed. Interestingly, in this cluster for occasional grandparental childcare propensity, the marginal effect on having a first birth turns positive and slightly significant (*p* < .05): that is, on average, the propensity of the grandparents to provide occasional childcare leads to a change in the probability of their adult children having a first birth that is equal to .1.

Figure 3 shows the predicted probability of a first-birth transition for different levels of grandparental childcare propensity for each country group and for each type of expected childcare provision (i.e., regular, occasional, or any type of childcare). The predicted values of grandparental childcare propensity are on the *x*-axis, and the predicted probability of experiencing a first-birth transition is on the *y*-axis. Regular grandparental childcare propensity has a negative (close to 0) but nonsignificant coefficient in the pro-egalitarian countries and a significant and positive coefficient in the pro-natalist countries: as the values of regular grandparental childcare propensity increase, the probability of having a first birth increases as well (up to .2).

The same pattern, albeit with lower rates, is observed in the pro-traditional countries. For occasional grandparental childcare propensity, the trends are the same in both the pro-traditional and the pro-natalist countries. In the pro-egalitarian countries, a positive association is found between occasional grandparental childcare propensity and fertility.

In summary, in the pro-egalitarian countries, the probability of having a first child is almost unaffected by an increase in the grandparental propensity to provide regular childcare. In these countries, the grandparents’ propensity to provide occasional childcare, which is most common in this context (Hank and Buber 2009), is positively and significantly associated with their adult children entering parenthood. In the pro-natalist countries, where there is a mix of public policies and family aid, the probability of having a first birth increases as the grandparental propensity to provide both occasional and regular childcare increases. Finally, in the pro-traditional countries, the probability of entering parenthood increases as both types of grandparental propensity to provide childcare (i.e., regular and occasional) increase. In this group, the predicted probabilities vary between .08 for very low levels and .17 for higher values of the grandparental propensity to provide childcare measures.

I also run several robustness checks. Specifically, I try other country clusters and implement cross-validation to check that the measurement error magnitude between the first and the second estimation step is negligible. Moreover, I run a pooled model and a single country model to examine the patterns in individual countries. I also run the model with different control variables in the second step and try different specifications for the first step. I run the model using logistic in the first step. Finally, in light of the debate about comparability across nonlinear models (Breen et al. 2018; Mood 2010), I run a pooled logistic model with an interaction between my main explanatory variable and the country groups, and I repeat the second step of the analysis using a linear probability model instead of a logistic model (Breen et al. 2018; Mood 2010). In all these robustness checks, the results are shown to be robust (see Tables A11 and A12 in the online appendix). Detailed explanations and the results can be found in section D of the online appendix.

## Discussion

The main contribution of this study is in providing a better understanding of the influence of potential grandparental childcare provision on the entry of adult children into parenthood. Previous studies have looked at this relationship by analyzing the importance of one characteristic at a time. This study is the first to consider the role of would-be grandparents from a multidimensional perspective. Parental influence is exerted through the capacity of parents to provide their children with emotional and monetary support as well as with investments of their time. To test my hypotheses, I built a measure of grandparental propensity to provide care given that the provision of childcare by would-be grandparents is, by definition, not observable. I opted for a two-step estimation approach using the first two waves of the SHARE. Second, given the importance of context in shaping individuals’ attitudes and preferences, I looked at cross-national differences by running separate models for pro-natalist, pro-traditional, and pro-egalitarian countries (Gauthier 1996). Furthermore, given the trade-off between the prevalence and the intensity of grandparental childcare provision across European countries (Hank and Buber 2009), I ran separate models for regular, occasional, and any type of grandparental childcare. This approach allowed me to capture the conceptualization of the family role and its interaction with family policies in each context.

I found that although grandparents play a role in their adult children’s entry into parenthood in every country studied, the relationship between grandparental childcare propensity and first-birth transitions varies according to the context. Specifically, I found that the expectation of regular grandparental childcare has a significant and positive effect in both the pro-natalist and the pro-traditional countries but a slightly negative and nonsignificant impact in the pro-egalitarian countries. For the grandparental propensity to provide occasional childcare, the pro-egalitarian countries have a positive and slightly significant coefficient. Specifically, the predicted probability of having a first birth increases as the grandparental propensity to provide occasional care increases.

A limitation of the data is that only half of the grandparents for each potential newborn, and for two consequent waves only, were observable. Given that maternal grandmothers are the main providers of grandparental childcare (Sear and Coall 2011), the effect of grandparental propensity might be underestimated for all of the male adult children. Furthermore, because some forms of detailed information on the adult children’s characteristics were either missing or inconsistent, it was difficult to explore potential moderator effects, such as those of socioeconomic status (i.e., income or education). Finally, and most importantly, several characteristics of the grandparents might change during the transition to grandparenthood. For example, a working would-be grandparent might decide to retire after learning that his or her adult child is pregnant (Van Bavel and De Winter 2013). Although most of these variables would lead to an underestimation of the grandparents’ role, others may lead to an overestimation. I ran further model specifications by alternatively dropping some of these variables, and the results (shown in online appendix D) were robust.

On a more speculative note, I argue that in every context, the expectation of grandparental childcare provision is a very important factor in an adult child’s decision to have a child. I can identify two reasons why this might be the case. First, from a micro-level psychological perspective, having a high propensity to look after their grandchildren implies that the parents are indirectly willing to look after their adult child. Sharing childcare responsibilities involves a certain level of physical contact between parents and their adult children and may enable the parents to offer psychological and emotional support to their adult children. In other words, the adult child is reassured and can consider the transition to parenthood with greater confidence. Second, from a macro perspective, the extent to which grandparents are perceived as playing a reassuring role varies across countries. I found that in the pro-egalitarian countries, which have supportive welfare regimes, entry into parenthood is influenced by the propensity of grandparents to provide occasional care only. In both the pro-traditional and the pro-natalist countries, which have weaker family policies, I found that grandparents have an impact on the first-birth transitions of their adult children depending on their propensity to provide both regular and occasional childcare. Differences in the historical development of the public policies of these countries may explain these diverging patterns. In the pro-traditional countries, the value of the family is well established, and the informal safety net is deeply rooted in society. Thus, in these countries, grandparents are expected to provide intensive levels of childcare (Hank and Buber 2009), in part to compensate for the lack of family-friendly public policies. As Mills et al. (2014:14) noted, “[The] perception of childcare may operate not only as a barrier to the wider use of childcare, but [as a] lack of momentum to create policies.” In pro-egalitarian countries, policies have always been driven by the goal of facilitating work-family reconciliation. In pro-natalist countries, policies have historically been aimed at boosting fertility (Luci-Greulich and Thévenon 2013; Thévenon 2011). Thus, in these countries, policies that support the reconciliation of work and family are fairly strong. However, these policies are more effective and responsive in the pro-egalitarian than in the pro-natalist cluster.

These cultural and institutional differences are linked to differences in fertility. In pro-traditional countries, where there is “too much family” (e.g., Italy), fertility is particularly low (Livi-Bacci 2001). According to Billari and Dalla Zuanna (2015), when the extended family exerts social pressure that leads prospective parents to develop excessive concerns about whether they can manage having children, they tend to delay entry into parenthood or forgo it altogether (Balbo and Mills 2011; Billari and Dalla Zuanna 2015), despite the familialistic orientation of the society. Furthermore, normative expectations about the intensity of grandparental childcare reduces the number of children the grandparents can look after. Adult children (and their siblings) might reduce their fertility accordingly.

By contrast, as Thévenon and Gauthier (2011) have argued, in pro-egalitarian countries, the implementation of family-friendly policies aimed at facilitating family-work reconciliation provide more effective support for fertility than the enactment of policies directly aimed at boosting fertility, as is done in pro-natalist countries. In pro-egalitarian countries, the coexistence of relatively strong systems of formal and informal care leads families to have more children because they perceive the formal system to be at least as reliable as the informal system. Consequently, as Herlofson and Hagestad (2012) have argued, grandparents in these countries are ready to intervene as caregivers in unexpected situations or when the need for care is exceptionally high; in other countries, grandparents help to raise their grandchildren in response to inefficiencies in the public childcare system. Finally, it is important to mention that in the pro-egalitarian countries, the propensity of grandparents to provide childcare has a positive impact on the fertility of their adult children for expected occasional childcare only, and is not related to the strength of the grandparent-grandchild relationship. This finding might suggest that in pro-egalitarian countries, grandparents have more opportunities to spend quality time with their grandchildren because they do not have to worry about basic caregiving.

In conclusion, this article contributes to our understanding of the complex relationship between the propensity of potential grandparents to provide childcare and their adult children’s transition to parenthood. Future research is required to better address the role of formal childcare *in conjunction* with that of informal care and how this relationship varies in different contexts. In some countries, such as those characterized by familialistic values, the cultural component of grandparenting might affect not only the adult children’s fertility but also the sustainability of grandparenthood. Access to informal childcare services—as a backup, and not as a substitute for public childcare—could ease the burden on grandparents while helping parents to reconcile work and family (Kaptijn et al. 2010; Thomese and Liefbroer 2013) and, ultimately, to achieve their desired family size.

## Electronic supplementary material

ESM 1 (PDF 1210 kb)

## Data Availability

SHARE data are open data; the link is provided in the manuscript.

## Footnotes

1 I also ran the model for any positive childcare provision. Because this latter specification results from the sum of both occasional and regular childcare provision, I do not directly refer to it in the hypothesis section.

2 Methodological debate remains about the comparability of nonlinear models across different samples (see, e.g., Breen et al. 2018; Mize et al. 2019; Mood 2010). Coefficients are not directly comparable because logistic models suffer from unobserved heterogeneity bias (Breen et al. 2018; Mood 2010). A first solution is to compute average marginal effects, which allows for comparison across groups and models (Mood 2010). A second solution is to use the predicted probabilities of having a first birth at different values of the main independent variable (Mize et al. 2019; Mood 2010)—in this case, grandparental propensity. These predicted probabilities are also comparable across samples. Although no clear consensus has been reached on how to best approach this issue, I use a very conservative interpretation of both average marginal effects and predicted probabilities at grandparental propensity values, focusing mainly on the within country results and comparing only signs across countries. In addition, I carry out several robustness checks that are commonly recognized as helpful throughout this strand of literature (see the online appendix for more details).

3 See Table A4 for occasional grandparental childcare provision, and see Table A6 for any type of grandparental childcare (both found in the online appendix).

4 Here, *t* – 1 represents the date of interview for the previous wave of the survey. Between *t* and *t* – 1, there could be a maximum interval of two years if the childbirth occurs the day before the interview.

5 Descriptive statistics are included in section C of the online appendix.

6 The grandparents are clustered within adult children.

7 ${\mathrm{GP}}_{f,i}^{\ast}={\upbeta}_0+{\hat{\upbeta}}_1{employment\ status}_{f,i,t-1}+\dots +{\hat{\upbeta}}_{\mathrm{n}}{voluntary\ activity}_{f,i,t-1}+{\upvarepsilon}_{i,f}.$ ${\hat{\upbeta}}_1,{\hat{\upbeta}}_2,\dots, {\hat{\upbeta}}_{\mathrm{n}}$ is the set of all the coefficients obtained in the first step. *Employment status*, . . . , *voluntary activity* are the actual values of those variables in the sample of would-be grandparents used in the second step. They are measured at *t* – 1.

8 See Table A9 in the online appendix for descriptive statistics on geographical distance distribution.
