# Supplementary material for: Counting on Potential Grandparents? Adult Children’s Entry Into Parenthood Across European Countries
Source: Demography. 2020 Jun 9;57(4):1393–414. doi: 10.1007/s13524-020-00890-8 (PMC7441078; doi:10.1007/s13524-020-00890-8)
Supplement: Supplementary file 1 — (PDF 1210 kb) [file 13524_2020_890_MOESM1_ESM.pdf]

ONLINE APPENDIX

Table of contents

A. Definition and construction of variables……………………………….…. p. 3

B. Additional Tables…………………………………………………….…… p. 6

Table A1: Descriptive Statistics for Second Step Control Variables.

Table A2: Results from First Step Estimation (Regular Grandparental Childcare)

Table A3: Results from Second Step Estimation (Regular Grandparental Childcare)

Table A4: Results from First Step Estimation (Occasional Grandparental Childcare)

Table A5: Results from Second Step Estimation (Occasional Grandparental Childcare)

Table A6: Results from First Step Estimation (Any Type of Grandparental Childcare)

Table A7: Results from Second Step Estimation (Any Type of Grandparental Childcare)

Table A8 Descriptive Statistics for Single Country Distribution in Each Country Cluster

Table A9: Descriptive Statistics for the First Step

Table A10: First Step Variables and mechanisms they are associated with

C. Additional Figures…………………………………………………... p. 23

Fig. A1: Predicted Regular Grandparental Propensity Distribution

Fig. A2: Predicted Occasional Grandparental Propensity Distribution

D. Sensitivity Analysis…………………………………………………. p.25

Fig A3: Predicted Probability Grandparental Childcare Propensity- Pooled Model

Fig A4: Predicted Probability Grandparental Childcare Propensity- Single-Country Model

Fig A5: Predicted Probability Grandparental Childcare Propensity- Saraceno-Keck Model

Fig A6: Predicted Probability Regular Grandparental Childcare Propensity- Sensitivity Analysis

Fig A7: Predicted Probability Occasional Grandparental Childcare Propensity- Sensitivity Analysis

Fig A8: Predicted Probability Any Type of Grandparental Childcare Propensity- Sensitivity Analysis

Fig A9: Difference in Median Estimates Between the Real Estimates and Their Predictions

Fig. A10: Predicted Probability Grandparental Childcare Propensity-Influential Cases

Table A11: Results from comparing the predictive margins (Logistic Pooled Model with Interaction between Grandparental Propensity and Group of Countries)

Table A12: Results from comparing the predictive margins (Linear Probability Model with Interaction between Grandparental Propensity and Group of Countries)
References…………………………………………………………………………......p. 37

A. Definition and construction of variables

Here, I provide more details about the definition of the first step variables. I group them into two big groups: namely, "general health" and "demographics."

A) GENERAL HEALTH

The aim of this set of variables is to measure different dimensions of grandparents' general health. I isolate three different groups:

1. Objective health, which includes cognitive, physical, and mental health measured by a brief medical test administered to the respondents;

2. Lifestyle, which refers to social activity (e.g., voluntary work) and healthy behaviors (e.g., smoking habits); and

3. Subjective health.

1) OBJECTIVE HEALTH

1.1. Cognitive health

- Numeracy score

This variable reflects the mathematical aptitude of the individual. The score is based on responses to questions linking mathematical reasoning or calculus with daily routine problem-solving. This is a categorical variable with values ranging from zero to five. Higher values correspond to higher performances.

- Orientation in time

This variable reflects the capacity of the respondent to orientate him/herself within time (based on answers to questions like "What day of the week/the month is it today?"). I recode it into a three-category variable: "good," "fair," and "bad."

- Recall ability

This variable reflects the respondent's short-term memory. The interviewer tests the respondent's memory by listing 10 words and asking him or her to repeat as many words as possible. The variable ranges from zero to 10, and it is considered as continuous.

- Verbal fluency score

This variable reflects the richness of the respondent's vocabulary. It is based on questions like "How many names of animals can you list?" The variable ranges from zero to 81, and is it is considered as continuous.

1.2. Physical health

- Grip strength

This variable reflects the respondent's strength when grasping an object. According to the medical literature, it is a very good predictor of a person's vitality and longevity. Since strength

is gender- and age-specific, different scales are applied to different gender and age groups. To create a homogeneous measure, the value for each individual was computed as the deviance from the score of a fictional standard individual belonging to the same age and gender group. It is considered as continuous in my specification.

- Physical limitations

This variable reflects the respondent's level of limitation in daily activities due to physical impairments. This measure is categorical and ranges from zero to three: severely limited; limited, but not severely; and not limited.

- Sports:

This is a categorical variable that refers to how frequently the respondent participates in sports activities.

1.3. Mental health

- Depression scale

This variable reflects the respondent's level of depression. It is measured by using the Euro D scale, which ranges from zero to 12, with higher values corresponding to higher depression levels. The variable is considered as continuous.

2) LIFESTYLE

2.1. Smoking

This variable reflects the respondent's smoking habits in the last 12 months. It is a categorical variable: "stopped smoking," "no daily smoking for at least one year," "and currently smoking."

2.2. Voluntary work/care of a disabled adult/partner

These are binary variables. They are equal to one if the individual performs each of them at least weekly, and zero otherwise.

3) SUBJECTIVE HEATLH

3.1. Self-perceived health

This variable is based on the respondent's answer to the question: "Would you say your health is…" The answers are classified according to five categories with ascending values: very good, good, fair, bad, and very bad. To make interpreting this variable easier, I reversed the scale so that the higher level was associated with better health. Furthermore, due to data constraints (i.e., some categories had too few values), I combined the last two categories (i.e., bad and very bad). It is possible that some selection drove this variable as individuals in poor health tend to be less likely to answer survey questions.

B) DEMOGRAPHICS

1.1. Grandparents’ employment status

This variable is built on the question: “In general, how would you describe your current situation?” The answer is categorized as follows: retired, employed, self-employed, unemployed, permanently sick or disabled, homemaker, and other (specify). Due to the very small sub-groups I recode this variable as follows:

- zero for the grandparents who were retired or homemakers;
- one for the grandparents who were employed or self-employed;
- two for the grandparents who were unemployed;
- three for all the other typologies (missing values were dropped because there were very few missing cases; i.e., less than the 0.001% of the sample).

The logic of this categorization was that both retired people and homemakers would have been less subject to time constraints than people who were working.

1.2. Marital status of the grandparent

This variable is a categorical variable; I distinguish between respondents who were living alone (because of widowhood or divorce) and respondents who were living with a partner (married or in a registered partnership).

1.3. Geographical distance

This variable reflects the distance between the household of the adult child and the place where his or her parents live, expressed in kilometers. The categories are co-resident, same building, between one and five kilometers, between five and 25 kilometers, and between 25 kilometers and more than 100 kilometers.

1.4. Area of residence

This variable distinguishes between parents living in a large town/city and parents living in a smaller town or a rural area. I included this variable because the type of relationship and the individuals’ roles may have differed substantially depending on the size of their place of residence.

B. Additional Tables

Table A1. Descriptive Statistics Second Step: Control Variables

	Pro-egalitarian		Protraditional		Pronatalist	
Adult child	Number	%	Number	%	Number	%
gender						
male	1428	56.7	2746	58.3	1171	57.7
female	1089	43.3	1964	41.7	860	42.3
birth cohort						
1958–1965	352	14.0	604	12.8	248	12.2
1966–1972	580	23.0	1109	23.5	393	19.4
1973–1977	718	28.5	1424	30.2	560	27.6
1978–1980	472	18.8	885	18.8	414	20.4
1980+	395	15.7	688	14.6	416	20.5
Total	2517	100.0	4710	100.0	2031	100.0
Grandparent						
gender						
male	820	47.5	1485	47.9	732	52.2
female	905	52.5	1612	52.1	670	47.8
birth cohort						
1901–1934	254	14.7	423	14.0	181	12.9
1935–1939	210	12.2	507	16.7	167	11.9
1940–1945	434	25.2	774	25.6	280	20.0
1945–1950	498	28.9	712	23.5	384	27.4
1950+	329	19.1	613	20.2	390	27.8
Total	1725	100.0	3029	100.0	1402	100.0

Table A2. Results from First Step Estimation (Dependent Variable: Regular Grandparental Childcare)

	Pro-egalitarian	Pro-traditional	Pro-natalist
female (ref=male)	0.027*	0.023	0.018
	[0.012]	[0.014]	[0.017]
single (ref. = living with a partner)	-0.012	-0.022	-0.033+
	[0.014]	[0.015]	[0.017]
employed/self-employed (ref.= retired)	-0.061***	-0.063***	-0.023
	[0.015]	[0.018]	[0.021]
unemployed	-0.032	-0.078*	-0.010
	[0.032]	[0.034]	[0.029]
other	-0.039	-0.068+	-0.018
	[0.027]	[0.037]	[0.037]
Gp_age	-0.004***	-0.005***	-0.004***
	[0.001]	[0.001]	[0.001]
n. of grandchildren (ref.=1)			
2	-0.001	0.027+	0.017
	[0.018]	[0.015]	[0.021]
3	0.023	0.062**	0.071**
	[0.021]	[0.019]	[0.026]
4	0.026	0.042*	0.099***
	[0.020]	[0.020]	[0.027]
5+	0.002	0.057**	0.075**
	[0.019]	[0.019]	[0.024]
n. of adult children (ref.=1)			
2	-0.132***	-0.243***	-0.249***
	[0.037]	[0.025]	[0.036]
3	-0.195***	-0.354***	-0.340***
	[0.037]	[0.026]	[0.037]
4	-0.226***	-0.422***	-0.412***
	[0.038]	[0.028]	[0.038]
Adult child employment status			
part-time\self-employed	0.084***	0.055**	0.020
	[0.015]	[0.018]	[0.019]
unemployed	0.002	0.000	-0.058*
	[0.028]	[0.029]	[0.029]
Other	-0.011	-0.026	-0.072***
	[0.014]	[0.016]	[0.020]
sex of adult child (ref. = male)	0.053***	0.118***	0.082***
	[0.010]	[0.012]	[0.013]
depression scale	0.003	-0.001	-0.003
	[0.003]	[0.003]	[0.004]
care for sick adult	0.016	0.066*	-0.013
	[0.024]	[0.026]	[0.022]
voluntary work	0.010	-0.003	0.003
	[0.015]	[0.024]	[0.020]
social activities	0.007	-0.002	0.000
	[0.012]	[0.019]	[0.019]
help to care spouse (ref=no)	-0.068*	-0.024	-0.100**
	[0.032]	[0.036]	[0.033]

Table A2 cont'd.

self-perceived health (ref. excellent)			
very good	-0.012	-0.024	0.008
	[0.017]	[0.026]	[0.026]
good	-0.034*	-0.015	0.004
	[0.017]	[0.025]	[0.025]
fair	-0.036	-0.016	-0.028
	[0.023]	[0.027]	[0.028]
poor	-0.050	0.004	0.023
	[0.036]	[0.036]	[0.043]
limited activities (ref.= severly limited)			
limited, but not severely	0.011	0.009	0.004
	[0.018]	[0.022]	[0.029]
not limited	0.016	0.008	-0.024
	[0.018]	[0.024]	[0.030]
Grip strength test	0.001	-0.021	0.077*
	[0.024]	[0.024]	[0.032]
Smoking (ref.= currently smoking)			
no daily smoking for at least one year	0.007	0.015	-0.015
	[0.013]	[0.015]	[0.020]
stopped smoking	0.026+	0.035*	-0.045*
	[0.014]	[0.017]	[0.021]
Sport (ref, = more than once a week)			
once a week	-0.002	-0.014	-0.000
	[0.015]	[0.016]	[0.021]
one to three times a month	0.001	-0.060**	0.005
	[0.019]	[0.020]	[0.026]
hardly ever, or never	-0.009	-0.005	-0.029+
	[0.013]	[0.014]	[0.017]
numeracy score (ref.= bad)			
2	-0.014	-0.005	0.018
	[0.054]	[0.025]	[0.030]
3	0.005	0.029	0.015
	[0.053]	[0.024]	[0.029]
4	0.003	0.004	0.011
	[0.054]	[0.026]	[0.030]
5 good	-0.015	0.009	0.039
	[0.054]	[0.029]	[0.035]
Orientation in time (ref.=bad)			
Fair	-0.101	-0.021	-0.157**
	[0.085]	[0.048]	[0.060]
good	-0.131	-0.026	-0.140*
	[0.085]	[0.045]	[0.058]
words listing	-0.001	-0.006+	0.003
	[0.003]	[0.003]	[0.004]
verbal fluency score	0.001	-0.000	0.001
	[0.001]	[0.001]	[0.001]
geographical proximity (ref.=coresident)			
less than 5 km	0.168***	0.297***	0.248**[illegible]
	[0.023]	[0.018]	[0.024]
between 5 and 25 km	0.050*	0.146***	0.114**[illegible]
	[0.022]	[0.020]	[0.023]
between 25 and 100 km	-0.005	-0.043+	-0.002
	[0.022]	[0.023]	[0.023]
more than 100 km	-0.062**	-0.113***	-0.090**
	[0.021]	[0.017]	[0.021]

Table A2 cont'd.

Area of building (ref= bigtown)					
town	0.001		0.020		-0.026+
	[0.012]		[0.014]		[0.016]
rural area	0.013		0.025		0.017
	[0.015]		[0.016]		[0.020]
country (ref.= Sweden)		country (ref.= Austria)		country (ref.= France)	
Netherlands	0.021	Germany	0.026	Belgium	0.021
	[0.014]		[0.021]		[0.015]
Denmark	-0.017	Spain	0.061*		
	[0.013]		[0.024]		
		Italy	0.102***		
			[0.024]		
		Greece	0.097***		
			[0.023]		
		Switzerland	0.044		
			[0.031]		
Constant	0.321**		0.328***		0.512***
	[0.107]		[0.071]		[0.087]
N	4520		5140		3670
R-sq	0.128		0.223		0.178

Standard errors in brackets + p<0.1, * p<0.05, ** p<0.01, *** p<0.001

Table A3. Results from Second Step Estimation (Dependent Variable: First Birth Transition- Regular Grandparental Childcare Propensity)

	Pro-egalitarian	Pro-traditional	Pro-natalist
grandparenting propensity	-0.022	0.746**	0.890*
	[0.461]	[0.262]	[0.390]
age (adult child)	-0.057+	-0.019	-0.034
	[0.032]	[0.028]	[0.043]
age (grandparent)	0.024	-0.037	-0.008
	[0.027]	[0.026]	[0.039]
adult child female (ref.=male)	0.277*	0.098	0.330*
	[0.109]	[0.096]	[0.137]
grandmother (ref=male)	0.019	-0.119	-0.058
	[0.113]	[0.101]	[0.142]
Adult child birth cohort (ref.= 1958-65)			
1966-72	0.322	0.239	0.099
	[0.326]	[0.247]	[0.395]
1973-77	0.003	-0.213	0.414
	[0.480]	[0.394]	[0.608]
1978-80	-0.862	-1.068*	0.008
	[0.594]	[0.498]	[0.750]
> 1980	-1.955**	-1.821**	-1.579+
	[0.700]	[0.605]	[0.891]
Grandparent birth cohort (ref.= 1901-34)			
1935-39	0.156	-0.230	0.204
	[0.336]	[0.235]	[0.446]
1940-45	0.337	-0.395	0.010
	[0.423]	[0.341]	[0.593]
1946-50	0.478	-0.529	-0.141
	[0.539]	[0.466]	[0.757]
> 1950	0.363	-0.854	-0.118
	[0.658]	[0.581]	[0.920]
Constant	-1452	1436	-0.549
	[2.336]	[2.161]	[3.361]
**N**	2517	4710	2031

Standard errors in brackets + p<0.1, * p<0.05, ** p<0.01, *** p<0.001

Table A4 Results from First Step Estimation (Dependent Variable: Occasional Grandparental Childcare)

	Pro-egalitaria	Pro-traditiona	Pro-natalist
female (ref=male)	-0.025+	0.004	-0.023
	[0.015]	[0.015]	[0.018]
single (ref. = living with a partner)	-0.004	0.010	0.013
	[0.017]	[0.015]	[0.019]
employed/self-employed (ref.= retired)	0.049*	0.065***	0.009
	[0.019]	[0.018]	[0.022]
unemployed	0.024	0.095*	-0.031
	[0.042]	[0.039]	[0.032]
other	-0.019	0.056	0.019
	[0.035]	[0.041]	[0.043]
Gp_age	-0.000	0.003**	-0.001
	[0.001]	[0.001]	[0.001]
n. of grandchildren (ref.=1)			
2	0.047*	-0.011	0.003
	[0.020]	[0.015]	[0.021]
3	0.103***	0.023	0.026
	[0.023]	[0.019]	[0.026]
4	0.159***	0.025	0.065*
	[0.023]	[0.021]	[0.028]
5+	0.175***	0.019	0.096***
	[0.023]	[0.020]	[0.025]
n. of adult children (ref.=1)			
2	-0.286***	-0.126***	-0.123***
	[0.039]	[0.026]	[0.036]
3	-0.356***	-0.151***	-0.183***
	[0.040]	[0.026]	[0.037]
4	-0.473***	-0.175***	-0.248***
	[0.041]	[0.029]	[0.039]
Adult child employment status			
part-time\self-employed	0.042*	0.049**	0.034
	[0.019]	[0.017]	[0.021]
unemployed	-0.063+	0.010	-0.013
	[0.037]	[0.023]	[0.034]
Other	-0.004	0.079***	0.025
	[0.021]	[0.016]	[0.023]
sex of adult child (ref. = male)	0.025+	0.001	0.008
	[0.015]	[0.011]	[0.015]
depression scale	-0.007+	-0.002	0.002
	[0.004]	[0.003]	[0.004]
care for sick adult	-0.024	-0.041	0.009
	[0.027]	[0.026]	[0.026]
voluntary work	0.025	-0.011	0.026
	[0.020]	[0.027]	[0.024]
social activities	0.014	0.001	-0.004
	[0.016]	[0.021]	[0.021]
help to care spouse (ref=no)	0.060	0.015	0.053
	[0.047]	[0.033]	[0.045]

Table A4 cont'd.

self-perceived health (ref. excellent)				
very good		0.012	0.019	-0.003
		[0.020]	[0.028]	[0.028]
good		0.004	-0.003	-0.002
		[0.020]	[0.027]	[0.027]
fair		0.028	-0.003	0.031
		[0.028]	[0.029]	[0.033]
poor		0.096+	-0.021	-0.039
		[0.050]	[0.037]	[0.049]
limited activities (ref.= severly limited)				
limited, but not severely		0.004	-0.010	-0.042
		[0.023]	[0.024]	[0.029]
not limited		0.003	0.013	0.012
		[0.024]	[0.025]	[0.030]
Grip strength test		-0.041	0.040+	-0.055
		[0.030]	[0.023]	[0.034]
Smoking (ref.= currently smoking)				
no daily smoking for at least one year		-0.005	-0.007	-0.006
		[0.017]	[0.016]	[0.022]
stopped smoking		-0.024	0.000	0.027
		[0.017]	[0.018]	[0.023]
Sport (ref, = more than once a week)				
once a week		0.002	0.017	-0.021
		[0.019]	[0.017]	[0.023]
one to three times a month		-0.009	0.045*	-0.006
		[0.024]	[0.022]	[0.030]
hardly ever, or never		0.003	0.008	-0.000
		[0.017]	[0.014]	[0.018]
numeracy score (ref.= bad)				
	2	-0.029	0.049*	0.019
		[0.065]	[0.023]	[0.036]
	3	-0.047	0.010	0.020
		[0.063]	[0.022]	[0.033]
	4	-0.018	0.041+	0.030
		[0.064]	[0.025]	[0.035]
5 good		-0.014	0.041	0.026
		[0.064]	[0.030]	[0.041]
Orientation in time (ref.=bad)				
Fair		0.013	0.004	0.107
		[0.092]	[0.046]	[0.066]
good		0.020	-0.004	0.094
		[0.091]	[0.043]	[0.063]
words listing		0.005	0.004	0.001
		[0.004]	[0.004]	[0.004]

Table A4 cont'd.

verbal fluency score	-0.002+		-0.001		-0.000
	[0.001]		[0.001]		[0.001]
geographical proximity (ref.=coresident)					
less than 5 km	0.231***		0.133***		0.211***
	[0.021]		[0.012]		[0.018]
between 5 and 25 km	0.331***		0.217***		0.286***
	[0.023]		[0.017]		[0.019]
between 25 and 100 km	0.299***		0.249***		0.355***
	[0.023]		[0.024]		[0.023]
more than 100 km	0.226***		0.240***		0.322***
	[0.023]		[0.020]		[0.025]
Area of building (ref= bigtown)					
town	0.018		-0.010		-0.013
	[0.015]		[0.015]		[0.018]
rural area	-0.009		-0.038*		-0.002
	[0.018]		[0.017]		[0.022]
country (ref.= Sweden)		country (ref.= Austria)		country (ref.= France)	
Netherlands	-0.032+	Germany	0.012	Belgium	-0.074***
	[0.017]		[0.022]		[0.017]
Denmark	0.023	Spain	-0.009		
	[0.018]		[0.025]		
		Italy	-0.078**		
			[0.024]		
		Greece	-0.069**		
			[0.025]		
		Switzerland	-0.021		
			[0.031]		
Constant	0.336**		0.140*		0.073
	[0.123]		[0.071]		[0.089]
N	4520		5140		3670
R-sq	0.081		0.088		0.085

Standard errors in brackets + p<0.1, * p<0.05, ** p<0.01, *** p<0.001

Table A5. Results from Second Step Estimation (Dependent Variable: First Birth Transition-Occasional Grandparental Childcare Propensity)

	Pro-egalitarian	Pro-traditional	Pro-Natalist
grandparenting propensity	0.787*	2.029***	1.991***
	[0.360]	[0.324]	[0.458]
age (adult child)	-0.060+	-0.028	-0.031
	[0.032]	[0.029]	[0.044]
age (grandparent)	0.025	-0.039	-0.006
	[0.027]	[0.026]	[0.040]
adult child female (ref.=male)	0.256*	0.069	0.272*
	[0.110]	[0.096]	[0.138]
grandmother (ref=male)	0.020	-0.039	-0.018
	[0.113]	[0.101]	[0.144]
Adult child birth cohort (ref.= 1958-65)			
1966-72	0.311	0.252	0.093
	[0.328]	[0.250]	[0.396]
1973-77	-0.007	-0.196	0.458
	[0.481]	[0.399]	[0.607]
1978-80	-0.857	-1.064*	0.149
	[0.594]	[0.503]	[0.753]
> 1980	-1.909**	-1.865**	-1.323
	[0.700]	[0.611]	[0.896]
Grandparent birth cohort (ref.= 1901-34)			
1935-39	0.160	-0.274	0.176
	[0.336]	[0.237]	[0.451]
1940-45	0.318	-0.463	-0.021
	[0.419]	[0.344]	[0.601]
1946-50	0.460	-0.606	-0.139
	[0.534]	[0.470]	[0.767]
> 1950	0.350	-0.936	-0.113
	[0.650]	[0.583]	[0.934]
Constant	-1.636	1.625	-1.235
	[2.302]	[2.162]	[3.346]
**N**	2517	4710	2033

Standard errors in brackets + p<0.1, * p<0.05, ** p<0.01, *** p<0.001

Table A6 Results from First Step Estimation (Dependent Variable: Any Type of Grandparental Childcare)

	Pro-egalitaria	Pro-traditiona	Pro-natalist
female (ref=male)	0.002	0.027*	-0.005
	[0.012]	[0.012]	[0.015]
single (ref. = living with a partner)	-0.015	-0.012	-0.021
	[0.014]	[0.013]	[0.016]
employed/self-employed (ref.= retired)	-0.011	0.002	-0.015
	[0.015]	[0.015]	[0.017]
unemployed	-0.008	0.017	-0.041
	[0.031]	[0.030]	[0.027]
other	-0.058*	-0.012	0.001
	[0.027]	[0.036]	[0.035]
Gp_age	-0.004***	-0.002+	-0.005***
	[0.001]	[0.001]	[0.001]
n. of grandchildren (ref.=1)			
2	0.046***	0.016	0.020
	[0.012]	[0.010]	[0.013]
3	0.126***	0.084***	0.097***
	[0.015]	[0.015]	[0.017]
4	0.186***	0.067***	0.164***
	[0.017]	[0.016]	[0.020]
5+	0.177***	0.077***	0.171***
	[0.016]	[0.016]	[0.018]
n. of adult children (ref.=1)			
2	-0.418***	-0.369***	-0.372***
	[0.016]	[0.014]	[0.016]
3	-0.551***	-0.505***	-0.523***
	[0.018]	[0.016]	[0.018]
4	-0.698***	-0.597***	-0.660***
	[0.020]	[0.020]	[0.021]
Adult child employment status			
part-time\self-employed	0.126***	0.104***	0.053**
	[0.018]	[0.018]	[0.020]
unemployed	-0.061	0.010	-0.070*
	[0.038]	[0.028]	[0.035]
Other	-0.015	0.053**	-0.047+
	[0.020]	[0.017]	[0.025]
sex of adult child (ref. = male)	0.078***	0.119***	0.090***
	[0.015]	[0.014]	[0.016]
depression scale	-0.004	-0.003	-0.001
	[0.003]	[0.003]	[0.003]
care for sick adult	-0.008	0.026	-0.004
	[0.020]	[0.020]	[0.022]
voluntary work	0.035*	-0.014	0.029
	[0.016]	[0.019]	[0.020]

Table A6 cont'd.

social activities	0.021	-0.001	-0.004
	[0.013]	[0.016]	[0.017]
help to care spouse (ref=no)	-0.007	-0.009	-0.047
	[0.033]	[0.030]	[0.037]
self-perceived health (ref. excellent)			
very good	-0.000	-0.004	0.005
	[0.016]	[0.021]	[0.022]
good	-0.030+	-0.018	0.002
	[0.016]	[0.020]	[0.020]
fair	-0.008	-0.019	0.003
	[0.023]	[0.022]	[0.025]
poor	0.045	-0.017	-0.016
	[0.039]	[0.031]	[0.039]
limited activities (ref.= severly limited)			
limited, but not severely	0.015	-0.001	-0.038
	[0.019]	[0.020]	[0.025]
not limited	0.018	0.021	-0.012
	[0.020]	[0.021]	[0.026]
Grip strength test	-0.040+	0.018	0.022
	[0.024]	[0.020]	[0.029]
Smoking (ref.= currently smoking)			
no daily smoking for at least one year	0.002	0.008	-0.021
	[0.013]	[0.013]	[0.017]
stopped smoking	0.002	0.035*	-0.018
	[0.014]	[0.015]	[0.019]
Sport (ref, = more than once a week)			
once a week	-0.000	0.003	-0.022
	[0.015]	[0.013]	[0.018]
one to three times a month	-0.008	-0.015	-0.002
	[0.018]	[0.016]	[0.022]
hardly ever, or never	-0.006	0.003	-0.029+
	[0.013]	[0.012]	[0.015]
numeracy score (ref.= bad)			
2	-0.043	0.044*	0.036
	[0.041]	[0.021]	[0.031]
3	-0.042	0.039+	0.035
	[0.040]	[0.020]	[0.029]
4	-0.015	0.046*	0.040
	[0.040]	[0.022]	[0.031]
5 good	-0.028	0.050+	0.066+
	[0.041]	[0.026]	[0.035]
Orientation in time (ref.=bad)			
Fair	-0.088	-0.016	-0.050
	[0.070]	[0.039]	[0.054]
good	-0.111	-0.030	-0.046
	[0.069]	[0.037]	[0.051]
words listing	0.004	-0.002	0.005
	[0.003]	[0.003]	[0.003]

Table A6 cont'd.

verbal fluency score	-0.001		-0.001		0.000
	[0.001]		[0.001]		[0.001]
geographical proximity (ref.=coresident)					
less than 5 km	0.400***		0.430***		0.459***
	[0.025]		[0.017]		[0.024]
between 5 and 25 km	0.381***		0.363***		0.400***
	[0.027]		[0.021]		[0.025]
between 25 and 100 km	0.293***		0.206***		0.353***
	[0.028]		[0.028]		[0.027]
more than 100 km	0.164***		0.126***		0.231***
	[0.027]		[0.023]		[0.029]
Area of building (ref= bigtown)					
town	0.019		0.011		-0.039**
	[0.012]		[0.012]		[0.015]
rural area	0.005		-0.013		0.014
	[0.015]		[0.014]		[0.017]
country (ref.= Sweden)					
Netherlands	-0.011	country (ref.=	0.039*	country (ref.=	-0.053***
	[0.014]	Germany	[0.018]	Belgium	[0.013]
Denmark	0.006		0.052*		
	[0.014]	Spain	[0.021]		
			0.024		
		Italy	[0.020]		
			0.027		
		Greece	[0.020]		
			0.023		
		Switzerland	[0.025]		
Constant	0.657***		0.468***		0.585***
	[0.088]		[0.059]		[0.071]
N	4520		5140		3670
R-sq	0.170		0.244		0.211

Standard errors in brackets + p<0.1, * p<0.05, ** p<0.01, *** p<0.001

Table A7. Results from Second Step Estimation (Dependent Variable: First Birth Transition - Any Type of Grandparental Childcare Propensity)

	Pro-egalitarian	Pro-traditional	Pro-natalist
grandparenting propensity	0.382	1.142***	1.185***
	[0.247]	[0.183]	[0.263]
age (adult child)	-0.059+	-0.025	-0.037
	[0.032]	[0.028]	[0.044]
age (grandparent)	0.026	-0.034	-0.001
	[0.027]	[0.026]	[0.040]
adult child female (ref.=male)	0.268*	0.079	0.290*
	[0.109]	[0.096]	[0.137]
grandmother (ref=male)	0.014	-0.074	-0.039
	[0.113]	[0.101]	[0.143]
Adult child birth cohort (ref.= 1958-65)			
1966-72	0.314	0.246	0.058
	[0.328]	[0.249]	[0.395]
1973-77	-0.002	-0.200	0.369
	[0.481]	[0.396]	[0.606]
1978-80	-0.861	-1.034*	0.045
	[0.594]	[0.501]	[0.749]
> 1980	-1.930**	-1.778**	-1.442
	[0.701]	[0.609]	[0.891]
Grandparent birth cohort (ref.= 1901-34)			
1935-39	0.154	-0.237	0.192
	[0.337]	[0.237]	[0.457]
1940-45	0.338	-0.405	0.020
	[0.420]	[0.344]	[0.610]
1946-50	0.485	-0.527	-0.094
	[0.535]	[0.470]	[0.778]
> 1950	0.376	-0.843	-0.069
	[0.653]	[0.584]	[0.947]
Constant	-1.663	1.143	-1.287
	[2.319]	[2.174]	[3.412]
**N**	2517	4710	2031

Standard errors in brackets + p<0.1, * p<0.05, ** p<0.01, *** p<0.001

Table A8 Descriptive Statistics for Single Country Distribution in Each Country Cluster

		Fisrt step	Second step
		N	N
Pro-natalist	Belgium	2125	1197
	France	1545	834
	**Total**	**3670**	**2031**
Pro-traditional			
	Austria	740	525
	Germany	1110	703
	Greece	903	1150
	Italy	957	1043
	Spain	1120	856
	Switzerland	310	433
	**Total**	**5140**	**4710**
Pro-egalitarian			
	Denmark	989	492
	Sweden	1834	968
	The Netherla	1697	1058
	**Total**	**4520**	**2518**

Table A9: Descriptive Statistics for the First Step, independent variables

	Pro-egalitarian		Pro-traditional		Pro-natalist	
	N	%	N	%	N	%
**Sex of grandparents**						
Male	1889	41.8	866	40	680	46.4
Female	2631	58.2	1299	60	784	53.6
**Marital status of grandparent**						
Living with partner	3572	79	3855	75	2712	73.9
Single	948	21	1285	25	958	26.1
**Grandparents type of employment**						
Retired	2719	60.2	4091	79.6	2568	70
Employed/self-employed	1443	31.9	818	15.9	829	22.6
Others	358	7.9	231	4.5	273	7.5
**Total n.of grandchildren**						
1	551	12.2	908	17.7	566	15.4
2	805	17.8	1270	24.7	674	18.4
3	634	14	802	15.6	489	13.3
4	698	15.4	719	14	459	12.5
5+	1832	40.5	1441	28	1482	40.4
**N. of adult children**						
1	160	3.5	352	6.8	212	5.8
2	1556	34.4	1908	37.1	1142	31.1
3	1512	33.5	1668	32.5	1224	33.4
4	1292	28.6	1212	23.6	1092	29.8
**Geographical proximity**						
Co-resident	175	3.87	718	13.97	338	9.21
Less than 5 Km	1579	34.93	2394	46.58	1263	34.41
Between 5 and 25Km	1129	24.98	1020	19.84	990	26.98
Between 25 and 100 Km	805	17.81	405	7.88	600	16.35
More than 100Km	832	18.41	603	11.73	479	13.05
**Area of building**						
Big town	1601	35.4	1399	27.2	1017	27.7
Town	2014	44.6	2404	46.8	1738	47.4
Rural area	905	20	1337	26	915	24.9
**Social activities**						
No	3379	74.8	4623	89.9	3074	83.8
Yes	1141	25.2	517	10.1	596	16.2
**Help to care spouse**						
No	4408	97.5	4985	97	3576	97.4
Yes	112	2.5	155	3	94	2.6
**Self-perceived health**						
Excellent	887	19.6	325	6.3	353	9.6
Very good	1066	23.6	895	17.4	780	21.3
Good	1731	38.3	2027	39.4	1611	43.9
Fair	692	15.3	1561	30.4	766	20.9
Poor	144	3.2	332	6.5	160	4.4
**Limited activities**						
Severely limited	630	13.9	418	8.1	372	10.1
Limited but not severely	1306	28.9	1801	35	912	24.9
Not limited	2584	57.2	2921	56.8	2386	65
**Smoking**						
Currently smoking	1107	24.5	871	16.9	603	16.4
No daily smoking	1788	39.6	3068	59.7	1974	53.8
Stopped smoking	1625	36	1201	23.4	1093	29.8
**Sport**						
More than once a week	2102	46.5	1819	35.4	1231	33.5
Once a week	713	15.8	860	16.7	554	15.1
One to three times a month	358	7.9	549	10.7	321	8.7
Hardly ever or never	1347	29.8	1912	37.2	1564	42.6
**Numeracy score**						
1 (bad)	69	1.5	432	8.4	192	5.2
2	520	11.5	1074	20.9	549	15
3	1559	34.5	1628	31.7	1221	33.3
4	1313	29	1450	28.2	1300	35.4
5 (good)	1059	23.4	556	10.8	408	11.1

Table A9 cont'd.

**Orientation in date**						
Bad	30	0.7	112	2.2	29	0.8
Fair	487	10.8	620	12.1	504	13.7
Good	4003	88.6	4408	85.8	3137	85.5
**Adult child employment**						
Full-time employed	2783	61.6	3105	60.4	2434	66.3
Part-time	924	20.4	890	17.3	646	17.6
Unemployed	156	3.5	239	4.6	185	5
Others	657	14.5	906	17.6	405	11
**Sex of the adult children**						
Male	2216	49	2478	48.2	1781	48.5
Female	2304	51	2662	51.8	1889	51.5
	**Mean value**	**s.d**	**Mean value**	**s.d**	**Mean value**	**s.d**
Age of grandparents	63.5	7.4	64.9	7.8	63.9	8
Depression scale	1.8	1.8	2.5	2.2	2.4	2.1
Grip strenght test (mean-centered values)	0.04	0.2	-0.03	0.3	0	0.2
Word listing	4.02	1.8	2.95	1.8	3.31	1.81
Verbal fluency score	22	6.3	17	8	20	6.5
**Total (N)**	**4520**	**100**	**5140**	**100**	**3670**	**100**

Table A10: First Step Variables and mechanisms they are associated with

Grandparents' characteristics	Health	Avalaibiliy	Willingness	Demographics
sex				x
marital status				x
employment status		x		
age	x	x		
n. grandchildren		x	x	
n. adult children		x	x	
depression	x			
care for sick adult		x		
voluntary work		x	x	
social activity		x	x	
help to care spouse		x		
self-perceived health	x			
limited activities	x			
grip strenght	x			
smoking	x			
sport	x	x	x	
numeracy score	x			
orientation in time	x			
word listing	x			
verbal fluency	x			
geographical proximity		x		
area of building				x

C. Additional Tables

Fig. A1 Predicted Regular Grandparental Childcare Propensity Distribution (Second Step Independent Variable)

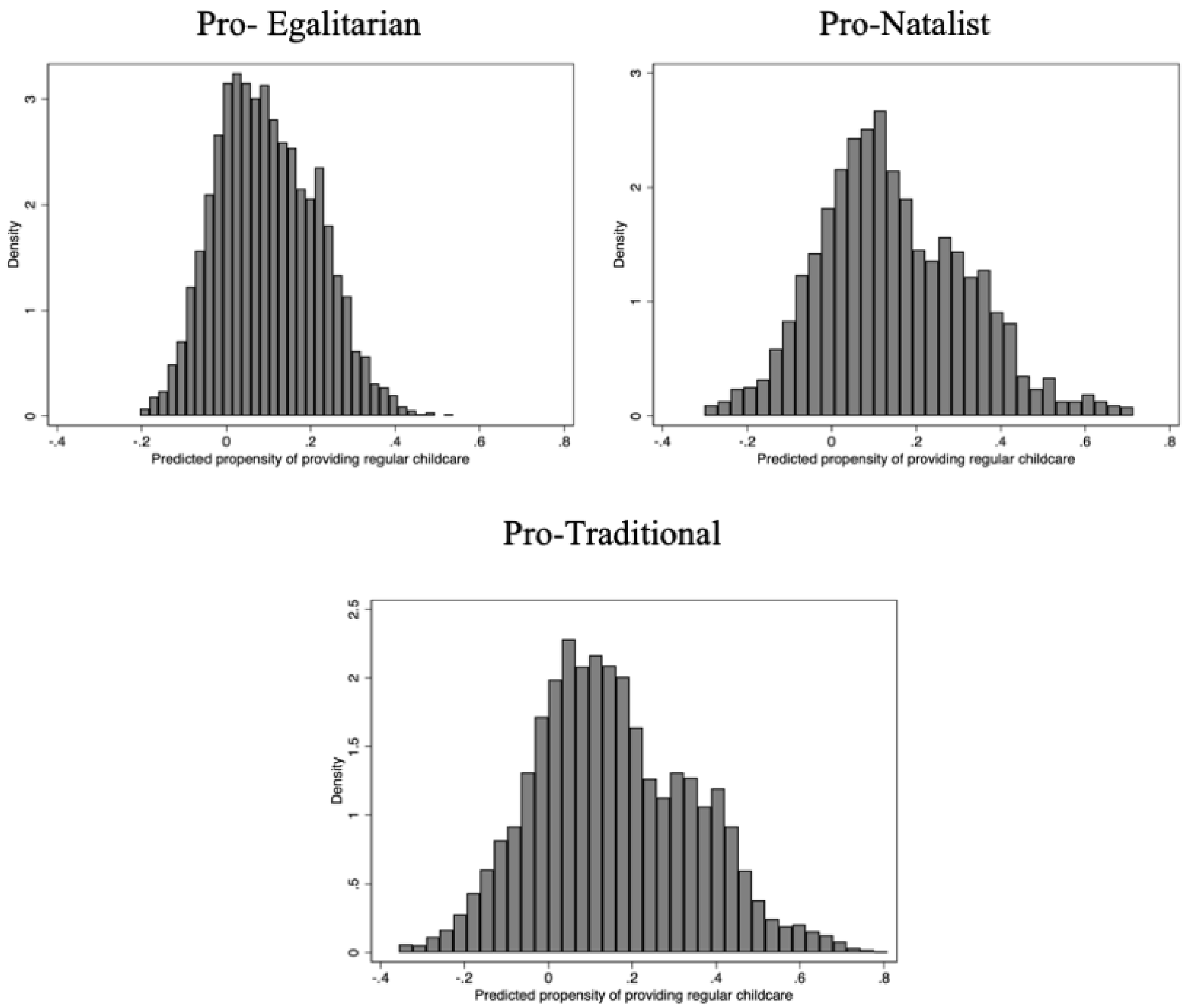

Fig. A2 Predicted Occasional Grandparental Childcare Propensity Distribution (Second Step Independent Variable)

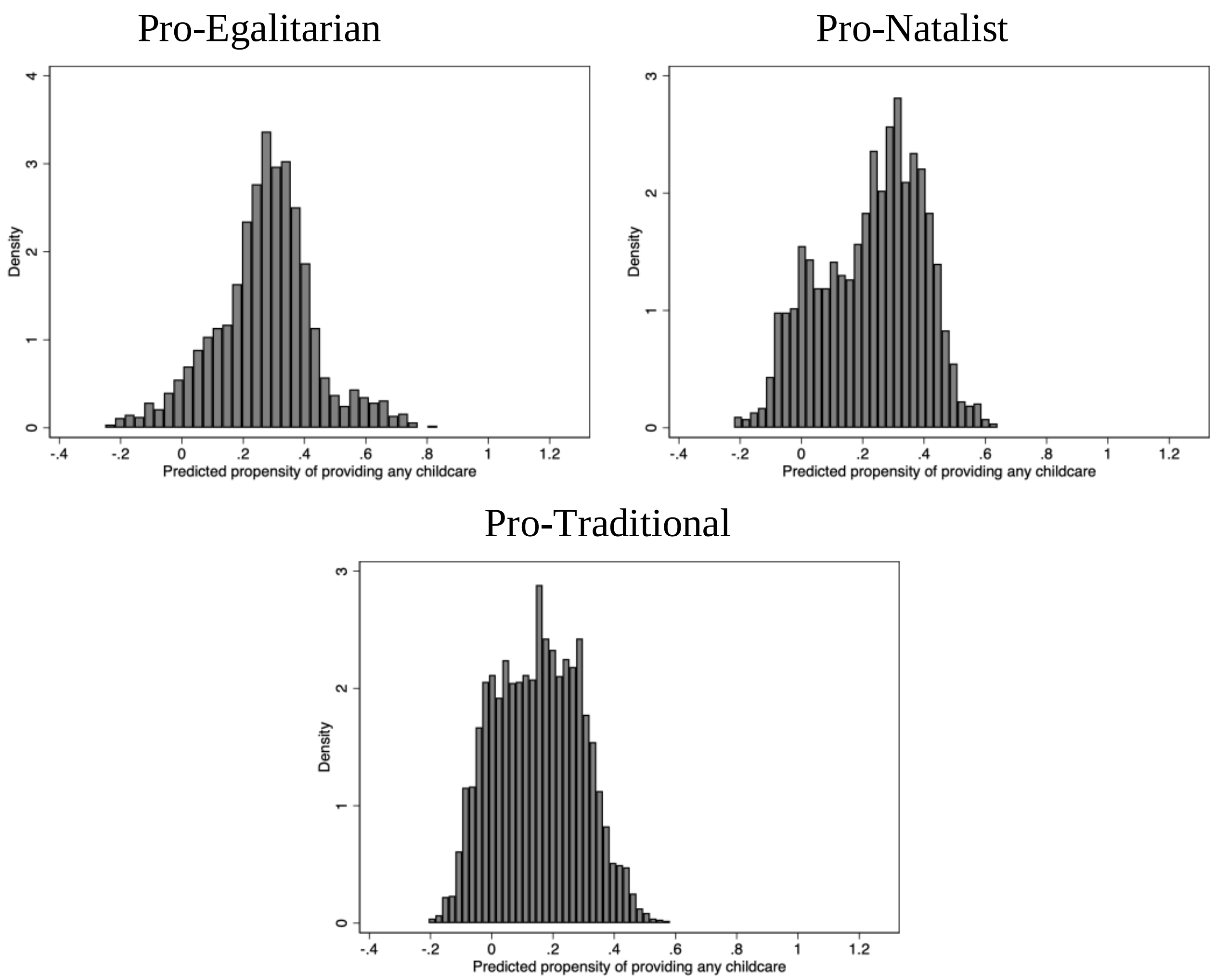

D. Sensitivity Analysis

To be sure that my results were sufficiently robust, I ran the following robustness checks. For the sake of clarity, in section D, I show only the predicted probabilities obtained in the second step estimation; every table and additional results as marginal effects are available upon request.

- I ran a pooled model including a dummy variable for each country, and found a strong positive effect for all types of grandparental propensity (see Fig. A3).
- I ran the model for each country separately (see Fig. A4). Fig. A4 shows the results of one country for each of Gauthier's clusters plus The Netherlands, which are in line with my main analysis. The analysis for regular grandparental propensity needs some attention. In the graph (A) of Fig. A4, the predicted probability for The Netherlands is slightly positive, although non-significant; whereas in the original results, the predicted probability for the pro-egalitarian group is slightly negative and non-significant.
- I cluster countries under alternative classifications that are sufficiently valid for the purposes of my research. Specifically, for each type of grandparental childcare provision, I ran my models in line with the classification provided by Saraceno and Keck (2010). In their system, country groups are formed according to the interplay between two different concepts: commodification/de-commodification (Esping-Andersen 1990 and 1999) and default/supported familialism/defamilialization (Leitner 2003; Saraceno and Keck 2010) (for more details, see Saraceno and Keck 2010; Keck and Saraceno 2011). The countries are clustered as follows: (1) weakly supported familialism-weak defamilialization (WSF-WD), in my sample: Greece, Italy, and Spain; (2) weakly supported familialism-strong defamilialization (WSF-SD), i.e., Denmark and Sweden; (3) strongly supported familialism-weak defamilialization (SSF-WD), i.e., Austria, Germany, and Switzerland; and (4) internally divergent (ID), i.e., Belgium, France, and The Netherlands.

  The results obtained after employing these categories for each type of grandparental propensity are in line with those I obtained using Gauthier's clusters (1996) (see Fig. A5). Focusing on the predicted probabilities of grandparental childcare propensity in the last estimation step, I found that in the WSF-SD group – which makes up part of Gauthier's pro-egalitarian cluster – the probabilities remain negative and not significant for regular grandparental propensity, whereas they turn positive in the occasional specification. For the WSF-WD group, which is a subgroup of Gauthier's pro-traditional countries, the predicted probability remains positive and the coefficients remain

significant for each type of grandparental propensity (i.e., regular, occasional, and any type), in line with my main findings. For the ID group, the predicted probabilities of regular grandparental childcare propensity are shown to be positive and the coefficients are found to be significant. I am aware that the ID group is not directly comparable with Gauthier's pro-natalist category because of my inclusion of The Netherlands. Finally, for the SSF-WD group, which is the other subgroup of Gauthier's pro-traditional cluster, the predicted probability is shown to be slightly negative for regular grandparental childcare propensity, although the coefficient is not significant. This outcome might also be related to the particularly small size of this subsample. For occasional and any type of grandparental childcare propensity, the predicted probability for the SSF/WD cluster turns positive, and the coefficient turns significant.

- Fig. A6, A7, and A8 show the predicted probabilities for different sensitivity analyses for the original model. Specifically, they display results for regular, occasional, and any type of grandparental propensity, respectively. In the first element of the graph matrix, I show the predicted probability from the specification I present in the main text (I called it "original"). This is followed by different graphs corresponding to different specifications, which I have identified with a capital letter on the top. Here, I provide descriptions of these different specifications.

  A: In light of the debate about whether a logistic model offers a better solution than a linear probability model, I ran the models using logistic in the first step.

  I tried to build alternative grandparental propensity scores by dropping in the first step some groups of variables that might bias the results through endogeneity.

  B: In the first step, the grandparental propensity is built by dropping all the variables related to physical health.

  C: In the first step, the grandparental propensity is built by dropping all the variables related to cognitive functions.

  D: In the first step, I kept only self-perceived health as a measure of both physical and cognitive health.

  E: I ran the models while dropping in the second step the variable "cohort," and leaving only linear age.

  F: I ran the model by adding the adult child's education level in the first step only; i.e., building the grandparental propensity.

  G: I ran the model adding the adult child's education level in both the first and the second step.

H: I ran the model adding the adult child's education level in the second step only. As we can see from the three figures (i.e., Fig. A6, A7, and A8), the results are very stable across different model specifications. This pattern holds for any type of grandparental propensity; i.e. regular, occasional, and any type.

- To check the predictive power of this two-step approach, I have predicted the grandparental propensity for actual grandparents. This allowed me to compare predictions with real values of grandparental childcare provision. Specifically, in the first step, I took one-half of my sample and trained the model on it. In the next step, I used the other half of that sample to predict my grandparental childcare propensity measure. I repeated this process 1,000 times (this number of iterations was based on the outcome of a formal test). I then ended up with a dataset in which I could compare 1,000 predictions and their corresponding 1,000 real values. I standardized my grandparental childcare propensity measure in order to set it between [0,1]. I computed the median value of the prediction for both values of the dependent variable (i.e., the median value of the prediction when DV=0 and a median value when DV=1). After computing the median values, I computed the difference between the dependent variable I predicted and the actual median value of the observations. For each value of the dependent variable, this difference in the median is fairly small (about 0.18 in the worst-case scenario. See Fig. A9).
- I ran a cross-validation analysis as well. I randomly divided the sample into five numerically equal subsamples. Next, for each of these subsamples, I used 80% of the data to train the model, and 20% of the data to test the model. I then repeated this five-subsample cross-validation technique 100 times (based on a rule of thumb for cross-validation), and computed the average RMSE (root mean square error). In my case, the average value of the RMSE was found to be around 0.4 for every group of countries. Smaller values of RMSE correspond to a better fit and a higher degree of reliability. I consider my result of 0.4 out of one acceptable.
- Due to the different sample sizes of single countries within each country cluster, I also ran some analyses of influential cases (i.e., Italy and Greece for pro-traditional and The Netherlands for pro-egalitarian). The results remain stable. The predicted probabilities for this specification are reported in Fig. A10 for each type of grandparental propensity.

Fig. A3 Predicted Probability of Different Types of Grandparental Childcare Propensity on Having a First Birth - Pooled Model. (A) Regular Grandparental Childcare Propensity (B) Occasional Grandparental Childcare Propensity (C) Any type of Grandparental Childcare Propensity

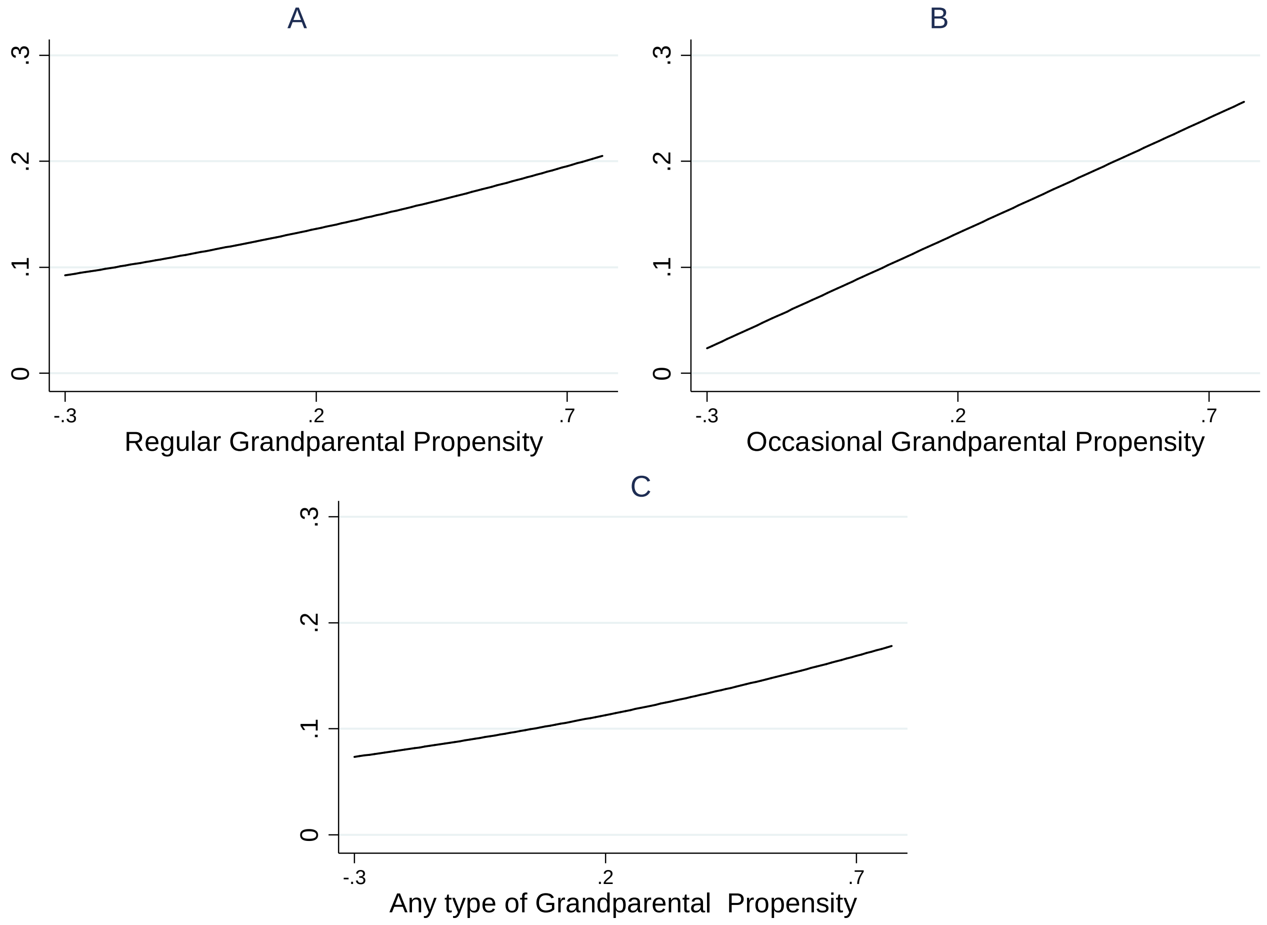

Fig. A4 Predicted Probability of Grandparental Childcare Propensity on Having a First Birth - Single Country Model. (A) Regular Grandparental Childcare Propensity (B) Occasional Grandparental Childcare Propensity (C) Any type of Grandparental Childcare Propensity

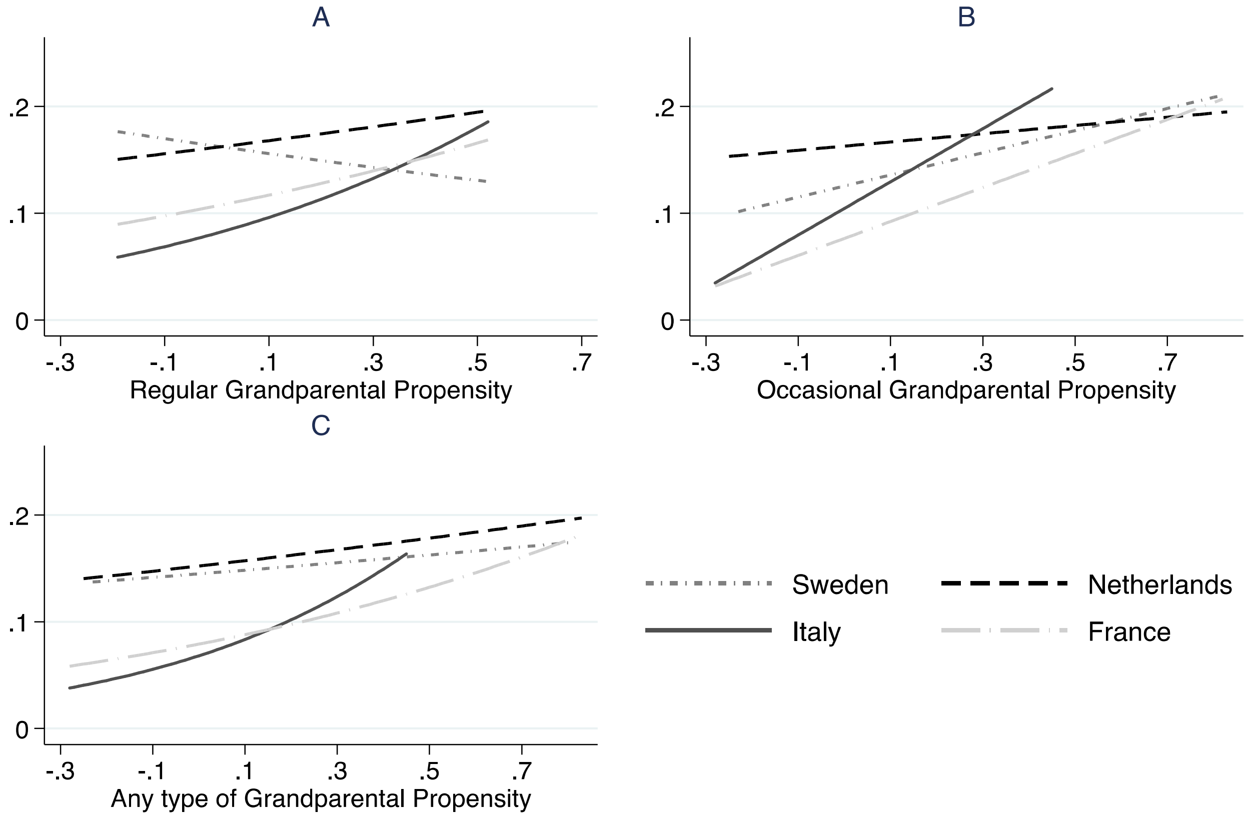

Fig. A5 Predicted Probability of Grandparental Childcare Propensity on Having a First Birth - Saraceno-Keck Country Clusters. (A) Regular Grandparental Childcare Propensity (B) Occasional Grandparental Childcare Propensity (C) Any type of Grandparental Childcare Propensity

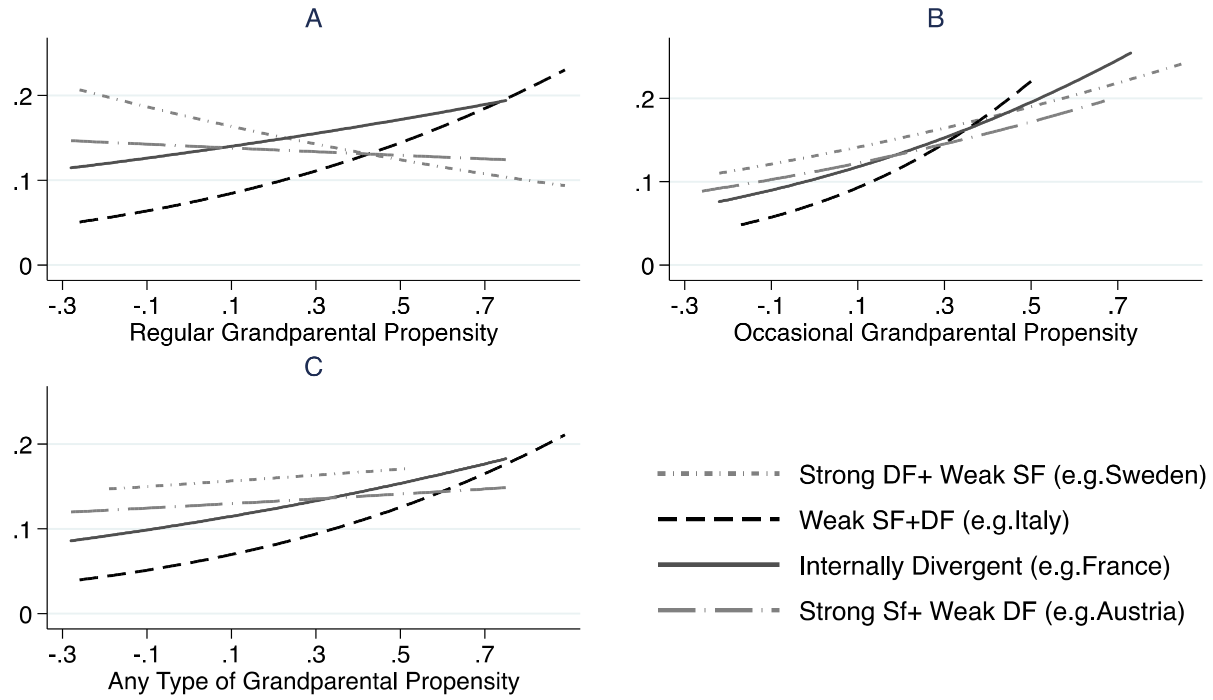

Fig. A6 Predicted Probability of Regular Grandparental Childcare Propensity on Having a First Birth - Second Step. Different model specifications

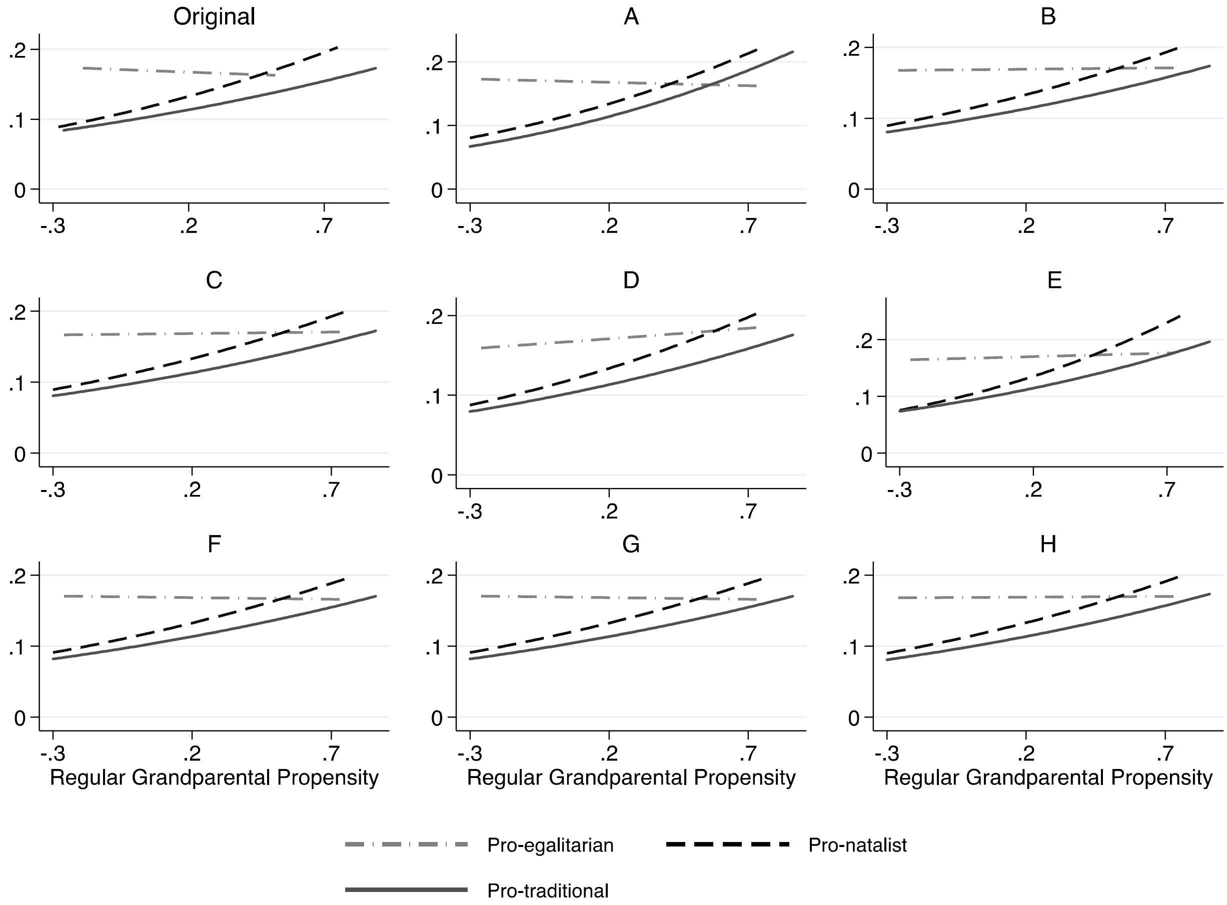

Fig. A7 Predicted Probability of Occasional Grandparental Childcare Propensity on Having a First Birth - Second Step. Different model specifications

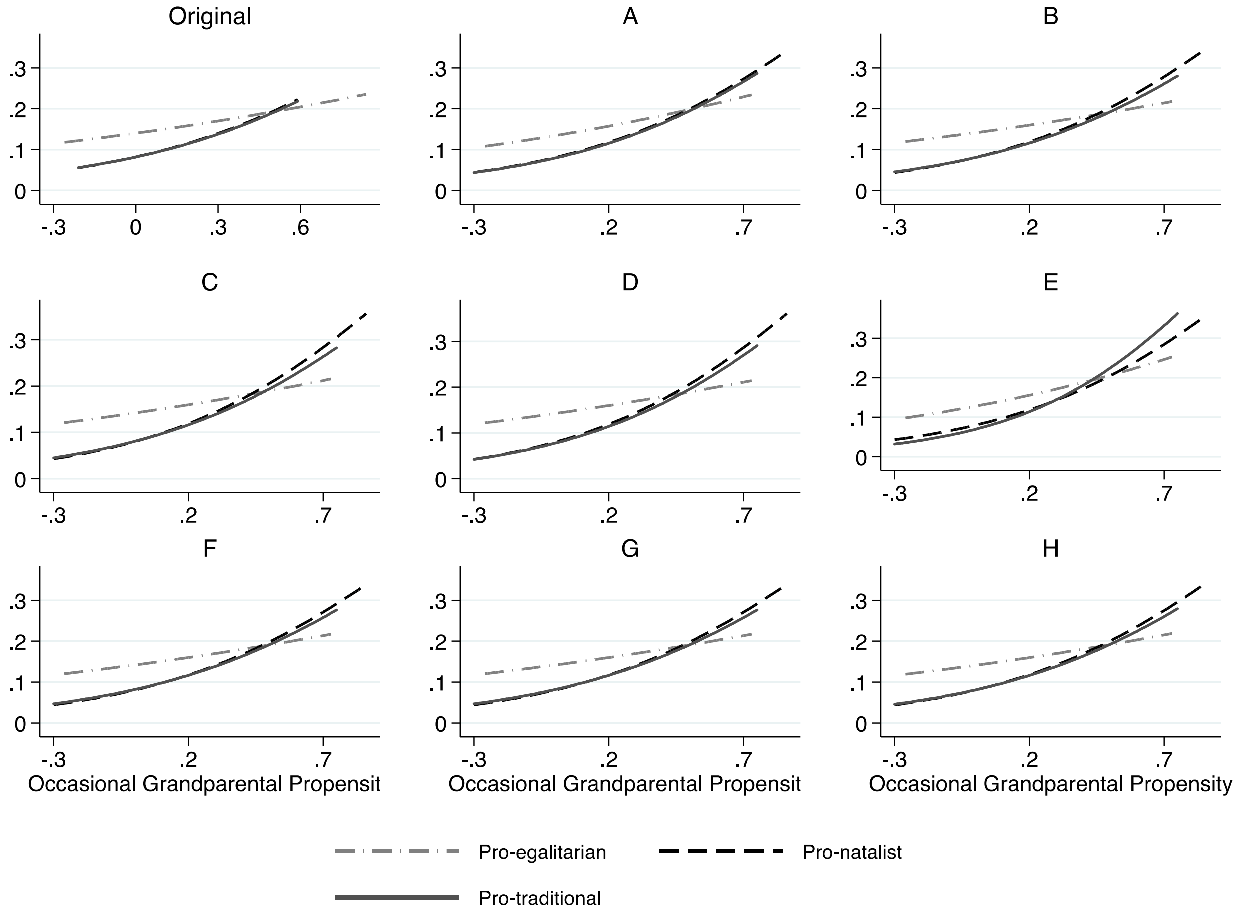

Fig. A8 Predicted Probability of Any Type of Grandparental Childcare Propensity on Having a First Birth - Second Step. Different model specifications

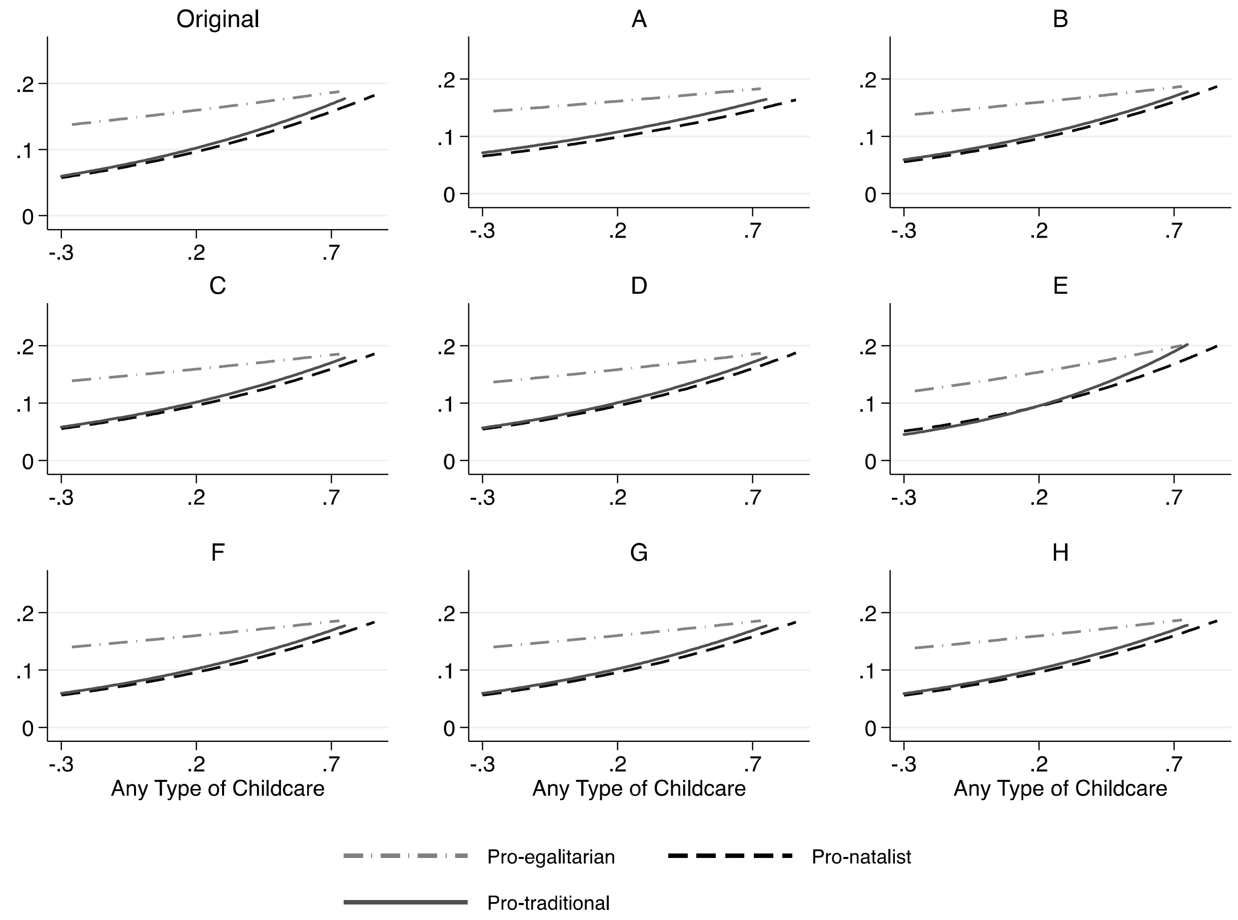

Fig. A9 Difference in Estimates Between the Real Estimates and Their Predictions. (1,000 iterations)

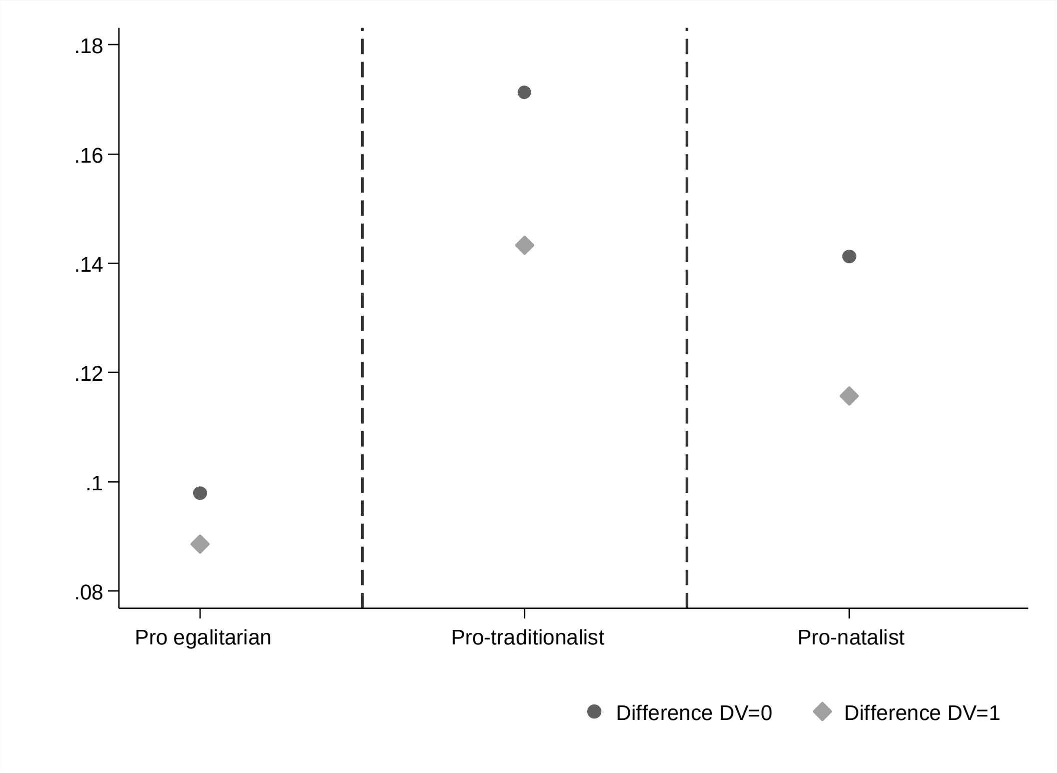

Fig. A10 Predicted Probability for Each Type of Grandparental Propensity on Having a First Birth - Second Step. Dropping potential influential countries

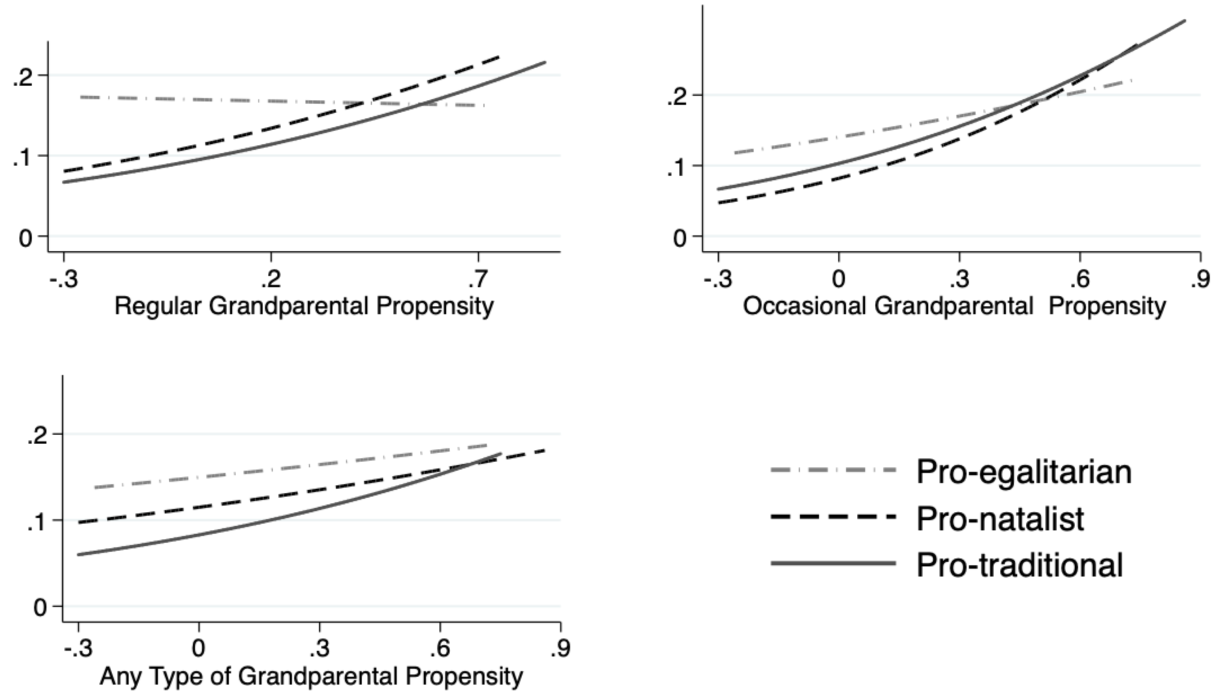

Table A11: Results from comparing the predictive margins adjusting for Bonferroni's method – Logistic model with interaction between grandparental propensity and group of countries

	Contrast	Std. Err	[95% Conf. Interval]	
Pro-traditional Vs Pro-egalitarian	-0.0584431	0.0090593	-0.0801309	-0.0367554
Pro-natalist Vs Pro-egalitarian	-0.0291324	0.0113447	-0.0562913	-0.0019735
Pro-natalist Vs Pro-traditional	0.0293108	0.009372	0.0068745	0.0517471

Table A12: Results from comparing the predictive margins adjusting for Bonferroni's method – Linear Probability model with interaction between grandparental propensity and group of countries

	Contrast	Std. Err	[95% Conf. Interval]	
Pro-traditional Vs Pro-egalitarian	-0.0595646	0.0091448	-0.0814631	-0.0376661
Pro-natalist Vs Pro-egalitarian	-0.0295927	0.0110054	-0.0559465	-0.0032388
Pro-natalist Vs Pro-traditional	0.029972	0.0090045	0.0084096	0.0515344

REFERENCES

Esping-Andersen, G. (1990). The Three Worlds of Welfare Capitalism.

Esping-Andersen, G. (1999). Social foundations of postindustrial economies. Oxford: Oxford University Press.

Breen, R., Karlson, K. B., & Holm, A. (2018). Interpreting and understanding logits, probits, and other nonlinear probability models. Annual Review of Sociology, 44, 39-54.

Keck, W., & Saraceno, C. (2011). Comparative childcare statistics in Europe Conceptual and methodological fallacies. Retrieved from http://www.carloalberto.org/assets/working-papers/no.229.pdf

Leitner, S. (2003). Varieties of familialism: the caring function of the family in comparative perspective. European societies, 5(4), 353–375.doi: 10.1080/1461669032000127642

Mize, T. D., Doan, L., & Long, J. S. (2019). A General Framework for Comparing Predictions and Marginal Effects across Models. Sociological Methodology, 0081175019852763.

Mood, C. (2010). Logistic regression: Why we cannot do what we think we can do, and what we can do about it. European sociological review, 26(1), 67-82.

Saraceno, C., & Keck, W. (2010). Can We Identify Intergenerational Policy Regimes in Europe? European Societies, 12(5), 675–696. https://doi.org/10.1080/14616696.2010.483006
